# Argyrophilic grain disease: epidemiology and association with cognitive decline and parkinsonism

**DOI:** 10.1093/braincomms/fcaf352

**Published:** 2025-10-13

**Authors:** Akira Arakawa, Tomoyasu Matsubara, Ayako Shioya, Manato Hara, Yuko Hiroyoshi, Masanori Kurihara, Satoru Morimoto, Takayuki Kato, Mana Higashihara, Renpei Sengoku, Masashi Kameyama, Kazutomi Kanemaru, Aya M Tokumaru, Tomio Arai, Norikazu Hara, Akinori Miyashita, Takeshi Ikeuchi, Airi Tarutani, Masato Hasegawa, Atsushi Iwata, Tatsushi Toda, Shigeo Murayama, Yuko Saito

**Affiliations:** Department of Neuropathology (the Brain Bank for Aging Research), Tokyo Metropolitan Institute for Geriatrics and Gerontology, Tokyo 173-0015, Japan; Department of Neurology, Graduate School of Medicine, The University of Tokyo, Tokyo 113-8655, Japan; Department of Neuropathology (the Brain Bank for Aging Research), Tokyo Metropolitan Institute for Geriatrics and Gerontology, Tokyo 173-0015, Japan; Department of Neuropathology (the Brain Bank for Aging Research), Tokyo Metropolitan Institute for Geriatrics and Gerontology, Tokyo 173-0015, Japan; Department of Neuropathology (the Brain Bank for Aging Research), Tokyo Metropolitan Institute for Geriatrics and Gerontology, Tokyo 173-0015, Japan; Department of Neurology, Graduate School of Medicine, The University of Tokyo, Tokyo 113-8655, Japan; Department of Neurology, Tokyo Metropolitan Institute for Geriatrics and Gerontology, Tokyo 173-0015, Japan; Department of Neurology, Tokyo Metropolitan Institute for Geriatrics and Gerontology, Tokyo 173-0015, Japan; Department of Neurology, Tokyo Metropolitan Institute for Geriatrics and Gerontology, Tokyo 173-0015, Japan; Department of Rehabilitation Medicine, Tokyo Metropolitan Institute for Geriatrics and Gerontology, Tokyo 173-0015, Japan; Department of Neurology, Tokyo Metropolitan Institute for Geriatrics and Gerontology, Tokyo 173-0015, Japan; Department of Neuropathology (the Brain Bank for Aging Research), Tokyo Metropolitan Institute for Geriatrics and Gerontology, Tokyo 173-0015, Japan; Research Team for Neuroimaging, Tokyo Metropolitan Institute for Geriatrics and Gerontology, Tokyo 173-0015, Japan; Department of Neurology, Tokyo Metropolitan Institute for Geriatrics and Gerontology, Tokyo 173-0015, Japan; Department of Diagnostic Radiology, Tokyo Metropolitan Institute for Geriatrics and Gerontology, Tokyo 173-0015, Japan; Department of Pathology, Tokyo Metropolitan Institute for Geriatrics and Gerontology, Tokyo 173-0015, Japan; Department of Molecular Genetics, Brain Research Institute, Niigata University, Niigata 951-8585, Japan; Department of Molecular Genetics, Brain Research Institute, Niigata University, Niigata 951-8585, Japan; Department of Molecular Genetics, Brain Research Institute, Niigata University, Niigata 951-8585, Japan; Department of Clinical Medical Sciences, Tokyo Metropolitan Institute of Medical Science, Tokyo 156-8506, Japan; Department of Clinical Medical Sciences, Tokyo Metropolitan Institute of Medical Science, Tokyo 156-8506, Japan; Department of Neurology, Tokyo Metropolitan Institute for Geriatrics and Gerontology, Tokyo 173-0015, Japan; Department of Neurology, Graduate School of Medicine, The University of Tokyo, Tokyo 113-8655, Japan; Department of Neuropathology (the Brain Bank for Aging Research), Tokyo Metropolitan Institute for Geriatrics and Gerontology, Tokyo 173-0015, Japan; The Brain Bank for Neurodevelopmental, Neurological and Psychiatric Disorders, United Graduate School of Child Development, Graduate School of Medicine, Osaka University, Osaka 565-0871, Japan; Department of Neuropathology (the Brain Bank for Aging Research), Tokyo Metropolitan Institute for Geriatrics and Gerontology, Tokyo 173-0015, Japan

**Keywords:** argyrophilic grain disease, granular-fuzzy astrocytes, parkinsonism, pretangles, substantia nigra

## Abstract

Argyrophilic grain disease is an age-related disorder characterized by the presence of argyrophilic grains. Argyrophilic grain disease has a sequential distribution pattern that begins in the ambient gyrus (Saito Stage I), spreads to the medial temporal lobe (Saito Stage II) and reaches the basal forebrain and cingulate gyrus (Saito Stage III). A strong association with cognitive decline, especially in cases of Saito Stage III argyrophilic grain disease, has also been reported. The main clinical feature includes cognitive decline characterized by memory disturbance, although conflicting results have been reported. Recent studies suggest an association with parkinsonism. To clarify the association between argyrophilic grain disease, cognitive decline and parkinsonism, we performed a clinicopathological study using the Brain Bank for Aging Research autopsy cohort in Japan. Approximately half (227) of the 452 consecutive autopsy cases had argyrophilic grain disease, and the frequency and stage of argyrophilic grain disease increased with age. Among the argyrophilic grain disease cases, 20 were demented without any comorbid pathology responsible for it, a condition referred to as dementia with grains. Furthermore, 6 of the 20 dementia with grains cases presented with parkinsonism, particularly postural instability, in addition to memory disturbance. Dementia with grains cases with parkinsonism had significantly more argyrophilic grains in the substantia nigra than those without parkinsonism and showed significantly decreased anti-dopamine transporter immunoreactivity in the putamen compared to control cases. Given these findings, argyrophilic grain disease is strongly associated with cognitive decline, especially in Saito Stage III cases, and parkinsonism is a new common clinical presentation. The extension of argyrophilic grain disease pathology to the nigrostriatal system may contribute to the development of parkinsonism.

## Teaser

Arakawa *et al*. report that argyrophilic grain disease is associated with cognitive decline, especially in the extensive Saito Stage III, and is also associated with parkinsonism. They also report that phosphorylated tau accumulation in the substantia nigra is associated with the presence of parkinsonism.

## Graphical Abstract

**Graphical Abstract:**
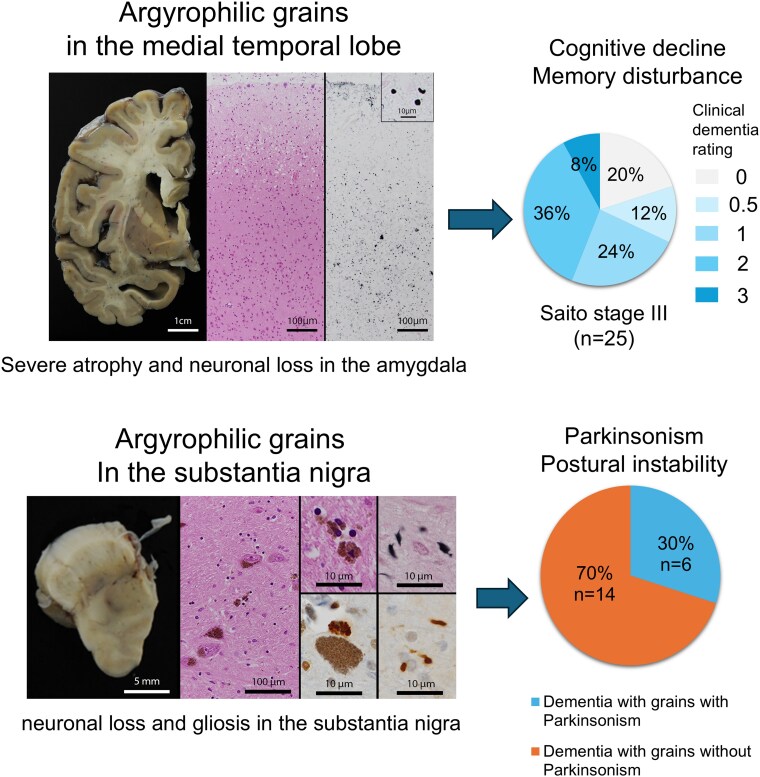

## Introduction

In 1987, Braak and Braak first reported argyrophilic grains with gradient-shaped or round Gallyas-positive structures.^1^ They defined argyrophilic grain disease (AGD) as a condition in which argyrophilic grains were observed in the brain. Argyrophilic grains primarily accumulated in the limbic system, especially in the ambient gyrus, entorhinal cortex and hippocampal CA1, and oligodendroglial coiled bodies were also observed in the affected areas.

Subsequent examination revealed that AGD is a four-repeat tauopathy,^2-5^ and other phosphorylated tau immunoreactive structures were observed: pretangles^6^ in the neuronal cytoplasm and bush-like astrocytes^7^ in the astrocytic dendrites, now called granular-fuzzy astrocytes (GFAs).^8^ Phosphorylated tau immunoreactive ballooned neurons were also identified.^9^ Studies using electron microscopy revealed that the argyrophilic grains were composed of many linear filaments with diameters of 9–19 nm.^10^ Western blotting using a phosphorylated tau antibody against the sarcosyl-insoluble fraction of the frozen brain showed a band pattern identical to corticobasal degeneration.^11^ Immunoelectron microscopy^12^ and Cryo-EM^13^ studies revealed that AGD has disease-specific structures of phosphorylated tau.

The most common clinical presentation of AGD in older adults is a slowly progressive memory disturbance.^2,14-19^ The term ‘dementia with grains (DG)’ was first used by Jellinger *et al*. to describe the presence of argyrophilic grains.^20^ Subsequently, we defined DG as a condition in which AGD is the sole pathology responsible for dementia.^14,21,22^ In 2004, we proposed an anatomical extension pattern from the ambient gyrus and amygdala (Saito Stage I) to the medial temporal lobe, including the transentorhinal gyrus and subiculum (Saito Stage II), and finally to the anterior cingulate gyrus, nucleus accumbens and basal forebrain (Saito Stage III). We also demonstrated a strong association with cognitive decline in Saito Stage III cases.^14^ We further identified 50 DG cases out of 1105 in our Brain Bank for Aging Research (BBAR) consecutive autopsy cohort (4.5%).^14^ Studies involving several consecutive autopsy cohorts supported this notion.^17,21,23^

However, studies involving other autopsy cohorts reported conflicting results. Several cohort-based studies from America, Europe and Brazil have found only a few DG cases, or even none.^24-27^ Recent studies have failed to demonstrate AGD as a risk factor for cognitive decline.^28-33^ Based on these reports, DG is considered a rare variant of AGD rather than a distinct disease entity. AGD is considered an additive pathology that lowers the threshold for cognitive decline in other comorbid pathologies. Whether AGD induces cognitive decline remains controversial.

Several case reports have suggested that patients with DG also present with parkinsonism.^34,35^ One autopsy cohort study reported several cases of AGD presenting with parkinsonism or gait instability.^26^ Another autopsy cohort study showed that AGD was the sole background pathology in 14.3% of cases with parkinsonism.^29^ Furthermore, a recent autopsy cohort study showed parkinsonism and severe nigrostriatal degeneration in two AGD cases with extensive pathology.^36^ We also report three cases of DG presenting with parkinsonism. Among them, two were diagnosed with progressive supranuclear palsy (PSP),^37,38^ and the other was diagnosed with Parkinson’s disease with dementia (PDD).^39^ However, the nigrostriatal degeneration was milder than that of neurodegenerative diseases, including Parkinson’s disease, PSP, corticobasal degeneration and multiple system atrophy, as well as the recently reported two AGD cases.^36^ The background pathology of parkinsonism or the prevalence of DG presenting with parkinsonism cases has not been fully elucidated to date.

In this report, we initially focus on the prevalence of AGD and its association with cognitive decline in the BBAR consecutive autopsy cohort, and then discuss the prevalence and clinical characteristics of parkinsonism observed in DG cases. Furthermore, we investigated the possible background pathologies of parkinsonism.

## Materials and methods

### Ethics statement

The brain samples used in this study were registered with the BBAR, with informed consent from the deceased’s relatives to carry out comprehensive research. The BBAR was approved by the Ethics Committee of the Tokyo Metropolitan Institute for Geriatrics and Gerontology. This study was performed in accordance with the tenets of the Declaration of Helsinki.

### Participants

The tissue samples were collected at the Tokyo Metropolitan Institute for Geriatrics and Gerontology, which provides community-based medical services to the elderly population. We obtained 452 consecutive brain autopsies (261 men and 191 women) from October 2012 to September 2022, after the observation period of our previous reports.^14,21^ Patient ages ranged from 24 to 111 years (mean ± SD, 79.3 ± 12.0).

### Clinical data

Clinical information was retrospectively obtained from medical charts and interviews with patients’ attending physicians and caregivers. The Clinical Dementia Rating (CDR) scale was used to grade cognitive decline, as previously reported, and dementia was defined as cases with a CDR score ≥1. The Mini-Mental State Examination (MMSE) and the revised version of Hasegawa’s dementia scale (HDS-R) were used to evaluate cognitive function. Among patients diagnosed with DG, we further examined neuropsychiatric symptoms, including irritability, dysregulation, apathy and delusion, based on medical records. Parkinsonism, including postural instability, ocular movements, rigidity, tremor and bradykinesia, was evaluated using the Unified Parkinson’s Disease Rating Scale (UPDRS) Part III^40^ on a 5-point scale from 0 (none) to 4 (severe). In addition, olfactory and autonomic dysfunctions, including orthostatic hypotension, urinary dysfunction and constipation, were retrieved from medical records.

### Neuropathology and immunohistochemistry

The brains and spinal cords were examined following BBAR protocols.^14,21,41-47^ Briefly, half of each brain was frozen for biochemical and molecular studies, and the other half was fixed for morphological studies. Most cases had autopsy imaging via CT and/or MRI during life. The more severe side was fixed, where asymmetric lesions were seen. When cerebrovascular lesions were present, the side was fixed. Otherwise, the fixed side was chosen randomly. The frozen hemisphere was sectioned into 7–8-mm slices in the coronal plane. The brainstem was sectioned into 5-mm slices in the axial plane, and the cerebellum was sectioned into 5-mm slices in the sagittal plane. The representative anatomical areas, including the anterior amygdala, posterior hippocampus and temporal pole, were sampled and fixed in 4% paraformaldehyde for two nights and embedded in paraffin. The other half was fixed in 20% buffered formalin (WAKO, Japan) for 7–13 days and sliced in the same manner. The representative areas were embedded in paraffin. Further, 6-μm-thick sections were stained using haematoxylin and eosin, Klüver–Barrera and Gallyas silver impregnation techniques. Subsequently, the immunohistochemically stained sections were visualized using a Ventana Bench-Mark GX autostainer (Ventana Medical Systems, Tucson, AZ, USA) with an I-View Universal DAB Detection Kit (Roche, Basel, Switzerland) and an I-View Universal Alkaline Phosphatase Red Detection Kit (Roche, Basel, Switzerland). Primary antibodies included anti-phosphorylated tau (mouse monoclonal, clone AT8; Innogenetics, Ghent, Belgium; 1:4000), anti-three-repeat tau (mouse monoclonal, clone RD3; Upstate, Lake Placid, NY, USA; 1:8000), anti-four-repeat tau (mouse monoclonal, clone RD4; Upstate, Lake Placid, NY, USA; 1:200), anti-phosphorylated-αSynuclein (mouse monoclonal, clone pSyn#64; a gift from T. Iwatsubo,^41^ now available for purchase from FUJIFILM Wako Pure Chemical Corp, Osaka, Japan; 1:20 000), anti-amyloid-β (mouse monoclonal, clone 12B2; IBL, Japan; 1:150) and anti-phosphorylated TAR deoxyribonucleic acid 43 kDa binding protein (TDP-43) antibodies (mouse monoclonal, clone 11-9; a gift from M. Hasegawa,^44^ now available for purchase from Cosmo Bio, Tokyo, Japan; 1:10 000).

### Staging of argyrophilic grains, the pathology of substantia nigra, and other neurodegenerative diseases and evaluation of vascular lesions

AGD staging in the limbic system was performed based on the Saito Stage^14,21,22^: Stage 0, none; Stage I, localized to the ambient gyrus and amygdala; Stage II, extending to the medial temporal lobe and posterior hippocampus; and Stage III, expanding to the lateral temporal lobe, septum, anterior cingulate gyrus and insular cortex, as determined by Gallyas staining. This evaluation was performed on both hemispheres, with the larger stage between the left and right sides evaluated as the representative stage of one case. The severity of the neuronal loss and gliosis in the substantia nigra was evaluated according to the following stages based on a recent report: Stage 0, no neuronal loss or gliosis; Stage 1, mild gliosis and neuronal loss; Stage 2, moderate neuronal loss and gliosis; and Stage 3, severe neuronal loss and fibrous gliosis, along with tissue rarefaction.^36^ The staging of argyrophilic grains in the substantia nigra was evaluated using AT8 immunostaining because melanin interferes with silver in the process of Gallyas staining. The semi-quantitative evaluation criteria were as follows: Stage 0, not observed; Stage 1, 1 or fewer per 20× objective field of view; Stage 2, 2–4 per 20× objective field of view; and Stage 3, 5 or more per 20× objective field of view. The number of pretangles and GFAs in the substantia nigra was evaluated semi-quantitatively using the same three stages with AT8 immunostaining.

For staging Alzheimer’s disease-associated amyloid β and phosphorylated tau pathology, we adopted the Braak senile plaque Stage,^48^ Consortium to Establish a Registry for Alzheimer’s Disease (CERAD) score,^49^ Thal senile plaque phase^50^ and Braak Neurofibrillary Tangle (NFT) Stage.^48,51^ For Lewy pathology, we evaluated the BBAR Lewy Stage.^41-43,45-47^ For phosphorylated TDP-43 (limbic-predominant age-related TDP-43 encephalopathy neuropathological change or LATE-NC), we evaluated the medial temporal lobe (amygdala and anterior hippocampus) of the fixed side using a three-step evaluation method (BBAR pTDP-43 Stage) as previously reported.^44^ When pTDP-43 cytoplasmic inclusions were observed (BBAR pTDP-43 stage ≥2), the other side was also examined. Hippocampal sclerosis was evaluated based on the presence of severe neuronal loss in the CA1 section in Ammon’s horn or the subiculum for both sides. Amyloid angiopathy was assessed according to the presence of amyloid β deposition in the cerebral vessels. Arteriolosclerosis was evaluated by the hyaline thickening of the walls of vessels <200 μm in diameter. Lacunars or microbleeds were identified based on the presence of infarcts or haemorrhages smaller than 5 mm in diameter in the entire brain sections.

### Extraction of pure AGD and diagnosis of DG

To diagnose DG cases, we first extracted ‘pure AGD’ cases from all AGD-positive cases. Among AGD-positive cases, we initially excluded those with other neurodegenerative diseases. We further excluded cases with other conditions that could cause cognitive decline and comorbid senile changes, including severe LATE-NC (>20 neuronal cytoplasmic inclusions per 20× objective field),^52,53^ moderate Braak NFT Stage (≥III) and prodromal Lewy body disease (BBAR Lewy Stage ≥ 2). The remaining cases were diagnosed as ‘pure AGD cases’, namely AGD-positive, Braak NFT Stage (≤II), minimal Lewy body pathology (BBAR Lewy Stage ≤1), and without severe LATE-NC or other neurodegenerative diseases or vascular lesions that could cause cognitive decline.^54^ DG was diagnosed when the CDR was ≥1 among pure AGD cases.

### Imaging findings of DG cases

MRI was performed on five of the DG cases with parkinsonism. Axial T2-weighted images and coronal fluid attenuated inversion recovery images were used for the visual assessment of the atrophy of medial temporal lobe and for the exclusion of gross lesions, including cerebral infarction. Further, diffusion-weighted images were used for the exclusion of acute cerebral infarction and T2* for the assessment of haemorrhage. For quantitative evaluation on MRI, voxel-based Specific Regional Analysis System for Alzheimer’s Disease (VSRAD) advance 2® was performed on T1-weighted images to assess atrophy in the limbic area and evaluate laterality,^55^ in addition to the size of the midbrain tegmentum on sagittal T1-weighted images.

Brain perfusion single-photon emission computed tomography (SPECT) using technetium-99-ethyl-cysteine dimer (ECD-SPECT) was performed for three cases, and dopamine transporter SPECT using ^123^I-ioflupane (DAT-SPECT) was conducted for two cases. For DAT-SPECT, the specific binding ratio (SBR) was used for evaluation. The SBR of striatal DAT binding was semi-quantitatively calculated using DAT VIEW software (Nihon Medi-Physics, Tokyo, Japan) and the Southampton method.^56^

### Semi-quantitative evaluation of anti-dopamine transporter staining

Positive findings on the anti-dopamine transporter antibody (rabbit monoclonal; Abcam, Tokyo, Japan; 1:250) immunostaining were quantified as follows: 10 locations per section were randomly photographed under a 20× objective field of view. Images were processed using Image J/Fiji software (NIH, Bethesda, MD, USA),^57^ and haematoxylin- and DAB-stained areas were segmented using the colour deconvolution function. After image binarization using the threshold function, the ratio of residual DAT-immunoreactive fibres was calculated as the DAT-immunoreactive area per capture area. In addition to the five DG cases with parkinsonism, the same staining and evaluation were performed on five other autopsied cases in which DAT-SPECT had been performed, and the accumulation was found to be within the normal range (DAT normal control). The densities were subsequently compared using the Mann–Whitney U-test.

### Tau immunoblotting and immunoelectron microscopy

Sarkosyl-insoluble tau extraction, electrophoresis and immunoblotting were performed as previously described.^11,12,36^ For immunoblotting, the membranes were incubated with primary anti-tau antibodies (T46, mouse, monoclonal, 1:1000, Thermo Fisher Scientific; tau-C: rabbit, polyclonal, 1:1000, Cosmo Bio). For immunoelectron microscopy, the grids were immunostained with an anti-C-terminal tau antibody (tau-C, 1:50).

### Genetic analysis of *MAPT* variants

Genetic analysis of *MAPT* variants was performed as previously described.^36^ Briefly, the genomic DNA was extracted from the middle frontal gyrus. The concentration was measured using the NanoDrop One^C^ (Thermo Fisher Scientific, Waltham, MA, USA). We used an Agilent 4200 TapeStation (Agilent Technologies, Santa Clara, CA, USA) for quality control and the BigDye Terminator v3.1 Cycle Sequencing Kit (Thermo Fisher Scientific) for Sanger sequencing.

### Statistical analysis

Statistical analyses were conducted using R version 4.0.3 (R Foundation for Statistical Computing, Vienna, Austria) and the graphical interface EZR (Saitama Medical Center, Jichi Medical University, Saitama, Japan).^58^ Differences in continuous variables, including pathological stages, were analysed using the Student’s *t*-test. Differences in ordinal variables were analysed using the chi-square test. Bias was adjusted using propensity scores. The Bonferroni correction was applied for multiple comparisons, and significance was set at *P* < 0.05. The results are expressed as the mean ± SD. To assess the effects of predictor variables on the CDR score, we performed a multivariable linear regression analysis using age, sex, Braak NFT Stage, Thal phase, Saito Stage, BBAR Lewy Stage and BBAR TDP-43 Stage as independent variables among cases aged ≥60 years. To evaluate the effects of predictor variables on the presence of dementia, we conducted a logistic regression analysis using the same independent variables among cases aged ≥60 years.

## Results

### Epidemiology of AGD

**Figure 1:**
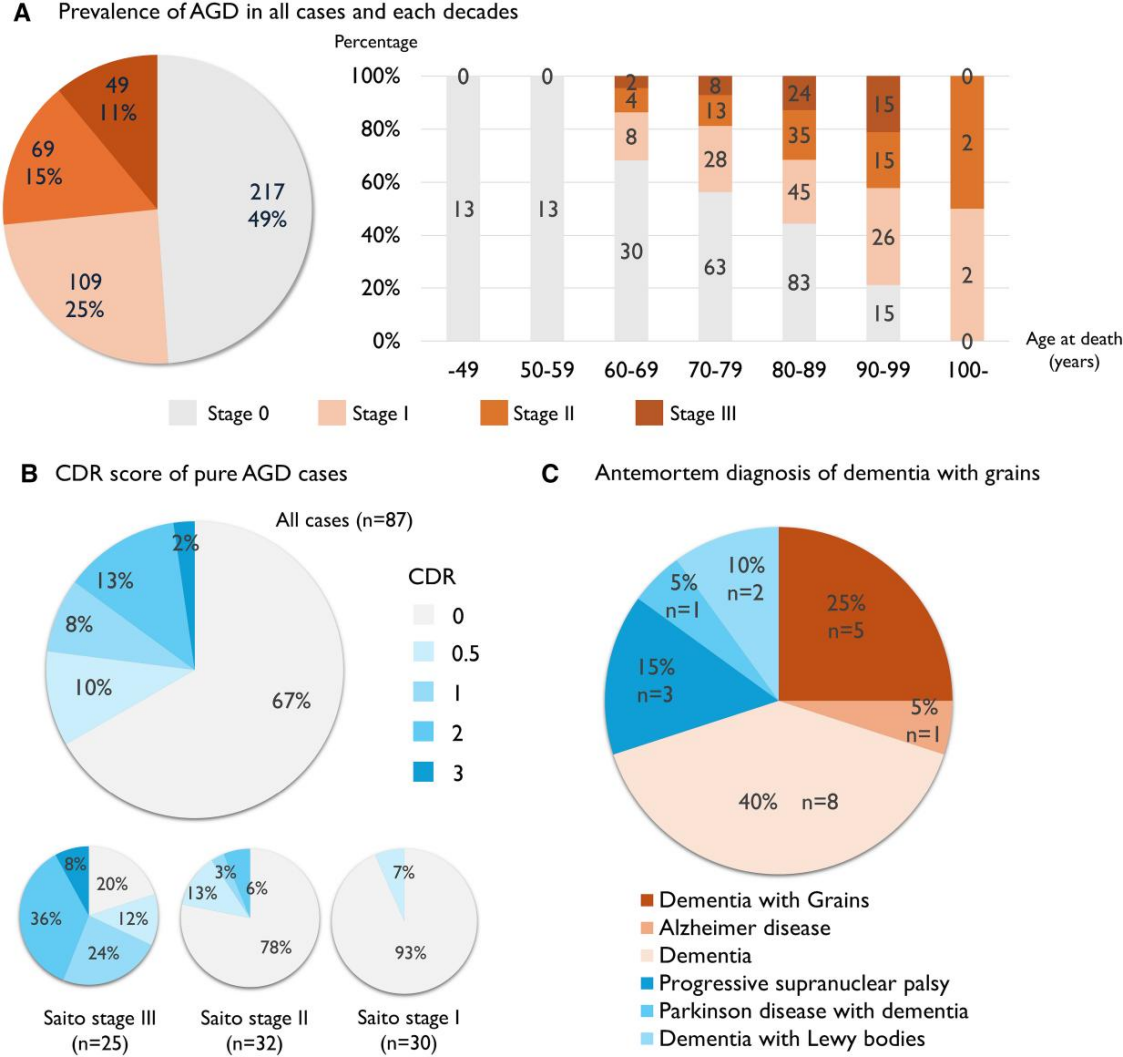
**Epidemiology of AGD.** (**A**) Approximately half of the patients had AGD pathology, and the prevalence rate increased with age. (**B**) Saito Stage III disease was strongly associated with cognitive decline. (**C**) 25% of DG patients had an antemortem diagnosis of DG. Furthermore, another 30% of patients were diagnosed with neurodegenerative diseases presenting with parkinsonism, including PSP, PDD and dementia with Lewy bodies. AGD, argyrophilic grain disease; CDR, Clinical Dementia Rating; DG, dementia with grains.

Among the 452 cases, 8 were excluded from the evaluation of the Saito Stage due to severe ischaemic changes or total brain necrosis. The remaining 444 cases included 257 males and 187 females, with an average age of 79.3 ± 11.9 years. We detected 227 (51.1%) AGD cases. Among them, 109 (24.5%) were classified as Saito Stage I, 69 (15.5%) as Saito Stage II and 49 (11.0%) as Saito Stage III. The frequency and stages of AGD increase with age. Among the patients aged <59 years, no AGD-positive cases were detected. In contrast, more than half (62.7%) of cases aged over 80 years were positive for AGD (Fig. 1A).

### Clinical aspects and comorbid pathology of AGD

We compared the rate of demented cases (CDR ≥ 1) and the average CDR score among the cases in each Saito Stage. The rate of demented cases and the average CDR score were significantly higher in AGD-positive cases (46.7% and 1.2 ± 1.2) than in AGD-negative cases (27.1% and 0.8 ± 1.2) (*P* < 0.001 and *P* = 0.006). The rate of demented cases and the average CDR score were significantly higher in Saito Stage III cases (77.6% and 1.7 ± 1.0) (*P* < 0.001 and *P* < 0.001) and in Saito Stage I cases (42.2% and 1.1 ± 1.2) (*P* = 0.0014 and *P* = 0.044). The rate and score tended to be higher, but with no significance, in Saito Stage II cases (31.9% and 0.8 ± 1.1). Among the cases in Saito Stage III and AGD-negative cases, the significance remained even when controlled for age, sex and comorbid Alzheimer pathology (40.5% versus 80.5% and 1.1 ± 1.4 versus 1.7 ± 1.1) (*P* < 0.001 and *P* = 0.028).

As for comorbid pathologies, Braak NFT Stage (2.4 ± 1.2 versus 1.7 ± 1.3) (*P* < 0.001) and the BBAR pTDP-43 Stage are significantly higher among cases with AGD compared to those without AGD (1.3 ± 1.1 versus 0.7 ± 0.9) (*P* < 0.001). However, the prevalence of Alzheimer’s disease cases (those categorized as ‘high’ in NIA-AA criteria^59^) is not significantly higher (7.9% versus 7.4%). The Thal phase and BBAR Lewy Stages are not significantly higher. The CERAD Stage was significantly, but only slightly, higher in AGD cases. The severity of these comorbid pathologies tended to increase with the AGD Stage (Table 1). In multivariable linear regression analysis, Braak NFT Stage, Saito Stage, BBAR Lewy Stage, BBAR TDP-43 Stage and age had independent effects on the CDR score. In logistic regression analysis, Braak NFT Stage, Saito Stage, BBAR Lewy Stage and BBAR TDP-43 Stage had independent effects on the presence of dementia (Supplementary Table 1).

**Table 1:** Clinical aspects and comorbid pathology of AGD

	AGD negative (n = 217)	AGD positive (*n* = 227)	Among each Saito stage cases			Age/sex matched cases		Age/sex AD matched cases	
			Saito Stage I (*n* = 109)	Saito Stage II (*n* = 69)	Saito Stage III (*n* = 49)	AGD negative (*n* = 46)	Saito Stage III (*n* = 46)	AGD negative (*n* = 42)	Saito Stage III (*n* = 42)
Age	74.9 ± 13.1	**83.9** **±** 8.5***	**83.3** **±** 9.2***	**84.4** **±** 8.5***	**84.6** **±** 7.1***	83.9 ± 6.8	83.9 ± 6.8	83.8 ± 7.5	83.7 ± 6.7
Sex (M/F)	133/84	124/103	59/50	37/32	28/21	24/22	26/20	28/14	25/17
Brain weight (g)	1237 ± 170.0	**1198** **±** 140.1**	1202 ± 147.4	1194 ± 140.1	1195 ± 125.3	1219 ± 130.1	1199 ± 126.1	1256 ± 119.0	1206 ± 125.2
Dementia (CDR≧1)	59 (27.1%)	106 (46.7%) ***	46 (42.2%) **	**22 (31.9%)**	38 (77.6%) ***	17 (44.7%)	37 (84.1%) ***	15 (40.5%)	33 (80.5%) ***
CDR	0.8 ± 1.2	**1.2** **±** 1.2**	**1.1** **±** 1.2*	**0.8** **±** **1.1**	**1.7** **±** 1.0***	1.1 ± 1.2	**1.7** **±** 1.0*	1.1 ± 1.4	**1.7** **±** 1.1*
CERAD	1.0 ± 1.1	**1.2** **±** 1.1*	1.2 ± 1.2	**1.3** **±** 1.1*	1.2 ± 1.1	1.3 ± 1.2	1.2 ± 1.2	1.2 ± 1.3	1.1 ± 1.1
Thal	2.0 ± 1.8	2.4 ± 1.7	2.4 ± 1.8	2.3 ± 1.6	2.2 ± 1.7	2.6 ± 1.9	2.2 ± 1.6	2.5 ± 1.9	2.2 ± 1.7
Braak NFT	1.7 ± 1.3	**2.4** **±** 1.2***	**2.5** **±** 1.4***	**2.3** **±** 1.1**	**2.3** **±** 0.9*	2.2 ± 1.2	2.3 ± 0.9	2.4 ± 1.1	2.3 ± 0.8
AD high cases	16 (7.4%)	18 (7.9%)	13 (11.9%)	3 (4.3%)	2 (4.1%)	4 (8.7%)	2 (4.3%)	4 (9.5%)	1 (2.4%)
BBAR Lewy	0.5 ± 1.1	0.6 ± 1.2	0.4 ± 1.3	0.7 ± 1.4	0.3 ± 0.8	0.9 ± 1.6	**0.3** **±** 0.8*	0.8 ± 1.3	**0.3** **±** 0.9*
BBAR pTDP43	0.7 ± 0.9	**1.3** **±** 1.1***	**1.2** **±** 1.1***	**1.1** **±** 1.1*	**1.5** **±** 1.2***	0.9 ± 1.0	**1.5** **±** 1.2*	1.1 ± 1.1	1.5 ± 1.2

AD, Alzheimer’s disease; AGD, argyrophilic grain disease; BBAR, Brain Bank for Aging Research; CDR, Clinical Dementia Rating; NFT, neurofibrillary tangle.

^*^ *P* < 0.05, **P < 0.01, ***P < 0.001, compared to AGD negative cases. Bold values indicate statistically significant differences.

### Extraction of pure AGD cases

Among the 227 AGD-positive cases, 83 had other neurodegenerative diseases, including Alzheimer’s disease (*n* = 21), NFT predominant dementia (*n* = 10), Lewy body disease (*n* = 18), frontotemporal lobar degeneration (*n* = 3), PSP (*n* = 9), corticobasal degeneration (*n* = 4), amyotrophic lateral sclerosis (*n* = 11), sporadic Creutzfeldt–Jakob disease (*n* = 4), multiple system atrophy (*n* = 1), spinocerebellar ataxia type 6 (*n* = 1) and atypical tauopathy (*n* = 1).

For the remaining cases, we further excluded vascular dementia (*n* = 9), brain tumours (*n* = 5), and 31 cases with other senile changes, including severe LATE-NC (*n* = 5), moderate Braak NFT Stage (≥III) (*n* = 21) and prodromal Lewy body disease (*n* = 5). The remaining 99 cases (41.8%) were diagnosed with pure AGD (Supplementary Table 2).

### Prevalence and antemortem diagnosis of DG cases

Among pure AGD cases, CDR data were available for 87 cases, and 20 (23%) cases were demented (CDR ≥ 1) and diagnosed as DG, constituting 4.5% of all cases. Furthermore, when including cases with CDR = 0.5, 29 (33%) had some cognitive decline. As for Stage III cases, 17 (68%) were demented, and 20 (80%) had some cognitive decline among 25 cases with available CDR data. Among Stage II cases, 3 (9%) were demented, and 7 (22%) had some cognitive decline among 32 cases with available CDR data. Among Stage I cases, no cases were demented, and two (7%) had mild cognitive decline (Fig. 1B).

Among the 20 DG cases, parkinsonism was observed in 6 cases (30%), irritability in 6 cases (30%), dysregulation in 3 cases (15%) and apathy in 2 cases (10%). Antemortem clinical diagnosis of DG was made in five cases (25%). Furthermore, the clinical diagnosis was PSP in three cases (15%), dementia with Lewy bodies (DLB) in two cases (10%) and PDD in one case (5%). The others were diagnosed simply as ‘dementia’ without thorough neurological examination, except for one case as Alzheimer’s disease (Fig. 1C). The diagnosis of AGD was made based on limbic-predominant atrophy on MRI (*n* = 4) with laterality (*n* = 1) and irritability (*n* = 1), in addition to slowly progressive memory disturbance. Only one case exhibited parkinsonism among the remaining 67 non-demented cases; however, drug-induced parkinsonism could not be ruled out due to the intake of sulpiride before the onset of parkinsonism.

### Clinical presentation of DG cases with parkinsonism

Among the six DG cases presenting with parkinsonism, the average age at death was 87.0 ± 4.3 years (range: 83–93 years). The disease duration was 5.2 ± 3.9 years (range: 2–11 years). The duration of cognitive decline was 3.0 ± 0.9 years (range: 2–4 years), and that of parkinsonism was 4.8 ± 3.7 years (range: 2–11 years). In two cases, parkinsonism preceded the onset of cognitive decline (7 and 6 years, respectively), and in the other two cases, cognitive decline preceded the onset of parkinsonism (1 year in both cases). The remaining two cases presented with parkinsonism and cognitive decline simultaneously. The CDR scores ranged from 1 to 2. The average scores were 17.5 ± 4.5 (range: 12–24) and 15.8 ± 5.2 (range: 7–21) on the MMSE and the HDS-R, respectively.

The predominant symptom of parkinsonism observed in all cases was moderate-to-severe postural instability [average UPDRS score 2.3 ± 0.5 (range: 2–3)]. Mild rigidity of the extremities was observed in four patients (UPDRS score 1). In two cases, parkinsonism was predominantly right-sided (Cases 4 and 5), while in the remaining four cases, it presented symmetrically. Mild tremors and akinesia were observed in two patients (UPDRS score 1, Cases 5 and 6 and Cases 1 and 6, respectively). Mild truncal rigidity was observed in one case (UPDRS score 1, Case 1). Symptoms commonly noted in Lewy body disease, including autonomic nervous system dysfunction, rapid eye movement sleep disorder and olfactory dysfunction, were not observed (Supplementary Table 3).

### Imaging findings of DG cases with parkinsonism

**Figure 2:**
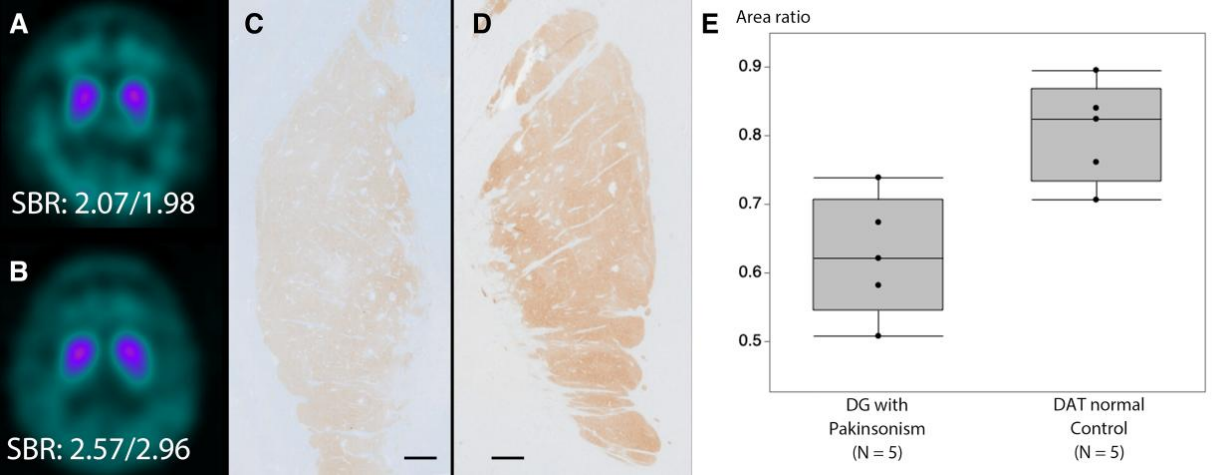
**DAT-SPECT findings and DAT immunostaining in patients with DG with parkinsonism.** (**A, B**) SPECT using ^123^I-ioflupane showing decreased uptake in the basal ganglia in Cases 2 (**A**) and 4 (**B**). The numbers below indicate the SBRs for the right and left sides. (**C, D**) Anti-dopamine transporter immunostaining showed decreased immunoreactivity in Case 4 (**C**) compared to the control (**D**). scalebar: 5 mm. (**E**) Anti-dopamine transporter immunostaining shows significantly lower immunoreactivity in DG cases with parkinsonism (*n* = 5) than in the DAT normal control (*n* = 5). *P* = 0.0159, as determined using Mann–Whitney U-test, is applied for the statistics. Each dot indicates an individual case. DG, dementia with grains; DAT, dopamine transporter; SBR, specific binding ratio; SPECT, single photon emission computed tomography.

Atrophy of the medial temporal lobe, including the amygdala and anterior hippocampus, was observed in all five cases, similar to that in typical AGD cases. Right-dominant atrophy of the medial temporal lobe was observed in four cases. The mean Z-score after quantitative analysis of the medial temporal lobe using VSRAD advance2® was 2.90 ± 1.13 (range: 0.81–4.12) on the right and 1.60 ± 1.08 (range: 0.51–3.58) on the left. The area of the midbrain tegmentum was 96.0 ± 8.4 mm^2^ (range: 83–107, normal range: 101–169^60^) (Supplementary Fig. 1). ECD-SPECT was performed in three cases, and decreased accumulation in the bilateral medial temporal lobes was observed. The accumulation in the posterior portion of the cingulate gyrus and praecuneus showed inconsistent results: one case (Case 1) showed decreased accumulation, another exhibited preserved accumulation (Case 3) and the third (Case 2) had slightly decreased accumulation. DAT-SPECT was performed in two cases and showed decreased uptake in the posterior putamen (SBR 2.07/1.98 for Case 2 and 2.94/3.28 for Case 4) (Fig. 2A and B).

### Neuropathological findings of DG cases with parkinsonism

Among the six DG cases presenting with parkinsonism, one was excluded due to a long postmortem interval (15 days in a freezer). Macroscopic examination revealed atrophy of the amygdala in all patients. Cases 1–3 showed moderate-to-severe atrophy, whereas Cases 4 and 5 showed mild atrophy. Depigmentation of the substantia nigra was observed in Case 1 but was very mild in Cases 2–5. Depigmentation of the locus coeruleus was not evident in any case. Atrophy of the limbic system was observed in the anterior hippocampus and amygdala but was not evident in the posterior hippocampus. Neocortical atrophy was not observed. The basal ganglia, thalamus, cerebellum and dentate nucleus showed no obvious atrophy or colour changes. No atrophy of the midbrain tegmentum or pons was observed. There was no evidence of macroscopic cerebrovascular disease in the basal ganglia of these patients (Supplementary Fig. 2).

**Figure 3:**
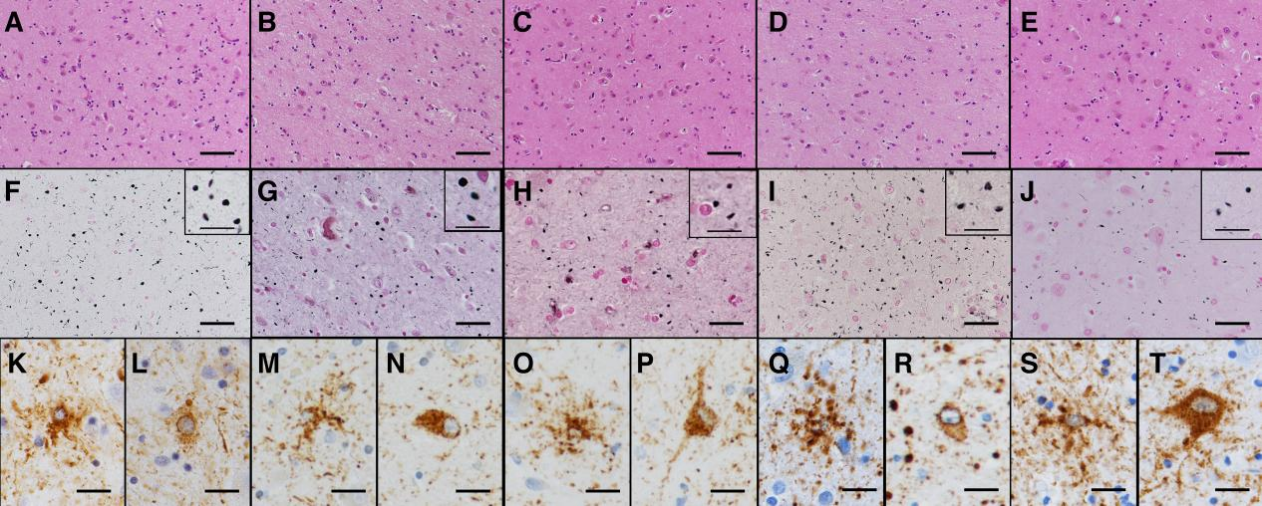
**Microscopic findings of the amygdala in patients with DG with parkinsonism.** (**A–E**) Neuronal loss and gliosis in the amygdala. (**F–J**) Large numbers of argyrophilic grains are observed. (**K, M, O, Q, S**) GFAs and (**L, N, P, R, T**) Pretangles were observed. (**A–E**) HE staining, (**F–J**) Gallyas staining and (**K–T**) AT8 immunostaining. (**A, F, K, L**) Case 1, (**B, G, M, N**) Case 2, (**C, H, O, P**) Case 3, (**D, I, Q, R**) Case 4 and (**E, J, S, T**) Case 5. Scalebars: (**A–E**) 200 µm, (**F–J**) 100 µm (inset: 20 µm) and (**K–T**) 20 µm. HE, haematoxylin–eosin.

Microscopic examination of the limbic system revealed neuronal loss and gliosis in the amygdala and ambient gyrus in all cases. Numerous argyrophilic grains were observed, and AT8 immunostaining revealed pretangles and GFAs (Fig. 3). Argyrophilic grains were also observed in the transentorhinal gyrus and subiculum, temporal pole, nucleus accumbens, septum and insular gyrus. The Saito Stage was (right/left) III/III in Case 1, III/II in Case 2, III/II in Case 3, III/I in Case 4 and I/II in Case 5, with right dominance in three cases and left dominance in one case. One patient did not exhibit laterality (Supplementary Table 4).

**Figure 4:**
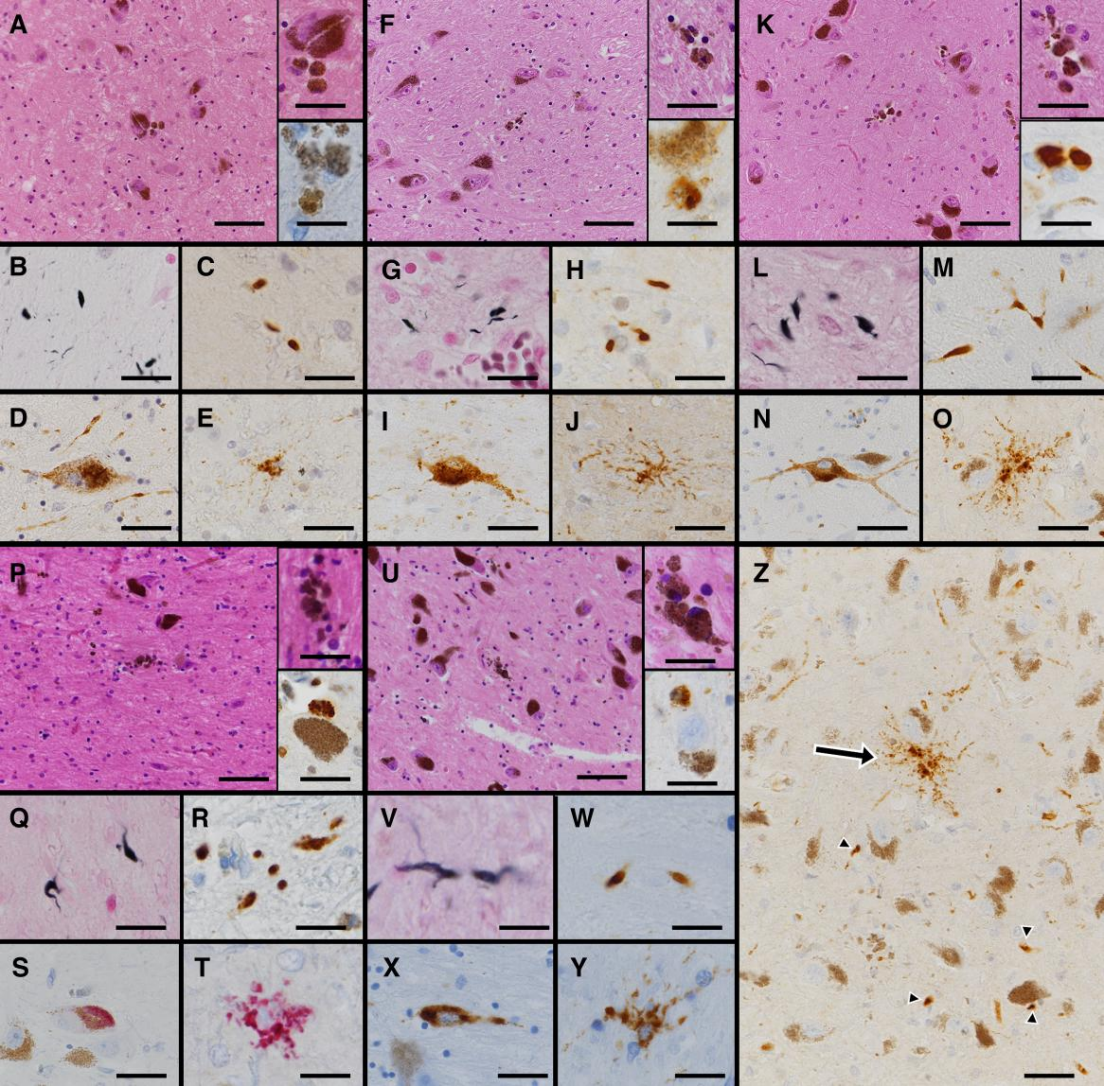
**Microscopic findings in the substantia nigra in patients with DG with parkinsonism.** (**A–E**) Case 1, (**F–J**) Case 2, (**K–O, Z**) Case 3, (**P–T**) Case 4 and (**U–Y**) Case 5. HE staining shows gliosis and melanin phagocytes (upper inset), and phagocytes containing four-repeat tau immunoreactive structures are observed (lower inset) (**A, F, K, P, U**). Gallyas-stain-positive grains (**B, G, L, Q, W**) and phosphorylated tau immunoreactive grains (**C, H, M, R, V**) were observed. Pretangles were observed in melanin-containing neurons (**D, I, N, S, X**) and GFAs (**E, J, O, T, Y**). Representative image of substantia nigra for Case 3, indicating granular fuzzy astrocytes (arrow) and phosphorylated tau immunoreactive grains (arrowheads) (**Z**). (**A, F, K, P, U**) HE staining (lower inset: RD4 immunostaining), (**B, G, L, Q, V**) Gallyas staining, (**C–E, H, I, M, N, R, W–Y**) AT8 immunostaining and (**S, T**) AT8 immunostaining with alkaline phosphatase red. (**J, O, Z**) RD4 immunostaining. Scalebars: (**A, F, K, P, U)** 100 µm (inset: 20 µm), (**B, C, G, H, L, M, Q, R, V, W**) 10 µm and (**D, E, I, J, N, O, S, T, X, Y, Z**): 20 µm. HE: haematoxylin–eosin.

In the substantia nigra, all five patients showed mild-to-moderate neuronal cell loss and gliosis. A few melanin phagocytes were observed, some of which were stained with RD4 immunostaining, while a few argyrophilic grains were observed with Gallyas, AT8 and RD4 immunostaining. A few GFAs and pretangles were observed (Fig. 4). A semi-quantitative evaluation of these findings is shown in Supplementary Table 4. Phosphorylated-TDP-43 immunoreactive structures were not observed in any of the cases. These AT8 immunoreactive structures were positive for RD4 but not for RD3.

There was no evidence of other tauopathies, such as tufted astrocytes, astrocytic plaques or globular glial inclusions, in the limbic system, basal ganglia, brainstem or neocortex. Other comorbid pathologies and microscopic vascular lesions are described in Supplementary Table 4.

On the anti-dopamine-transporter immunostaining in the putamen, the mean positive finding/area ratio after quantification in Cases 1–5 (0.63 ± 0.09, range: 0.51–0.74) was significantly lower than that in the DAT normal control group (0.81 ± 0.07, range: 0.71–0.90) (*P* = 0.0159) (Fig. 2C–E). The clinical diagnosis of DAT normal control included two Alzheimer’s disease cases without parkinsonism, one spinocerebellar ataxia type 1 case without parkinsonism and two cases with drug-associated parkinsonism. The age, brain weight and comorbid pathologies of DG cases with parkinsonism and DAT normal control cases are described in Supplementary Table 5.

### Western blotting, immunoelectron microscopy and genetic analysis

**Figure 5:**
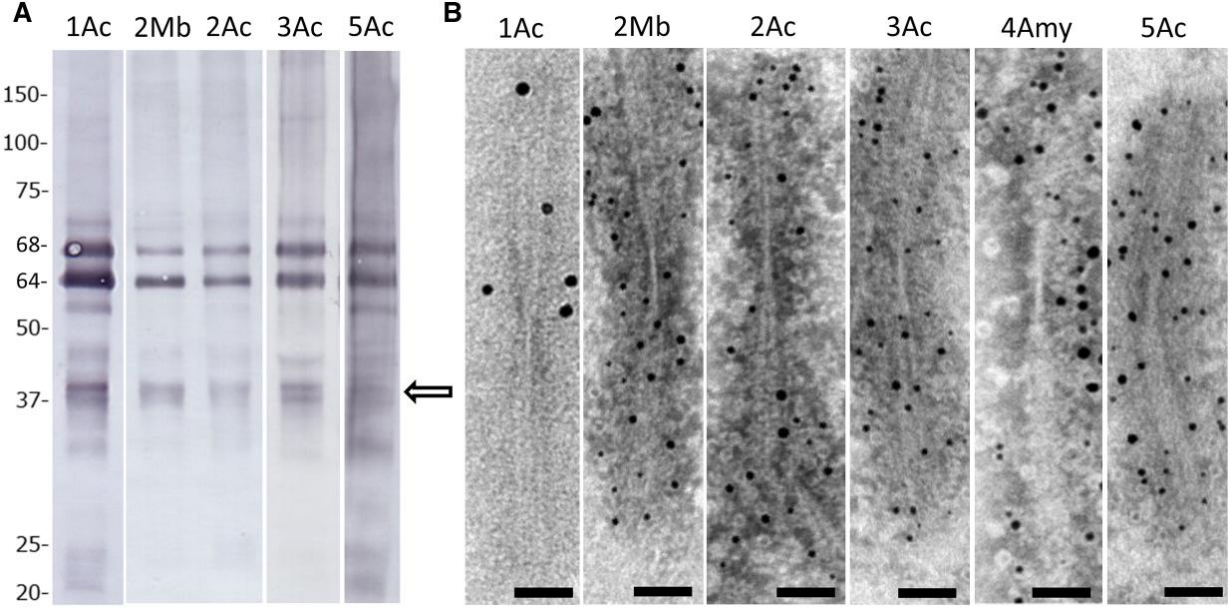
**Western blotting and immunoelectron microscopy in patients with DG with parkinsonism.** (**A**) Western blot of sarkosyl-insoluble fractions extracted from five AGD cases using C-terminal tau antibodies. Doublet bands were observed at ∼37 kDa (arrow) in addition to the 68 and 64 kDa bands of full-length four-repeat tau. (Cases 1–3: T46 antibody and Case 5: tau-C antibody) The picture of uncropped blots/gels is shown in Supplementary Fig. 3. (**B**) Immunoelectron microscopy of the sarkosyl-insoluble fractions extracted from 5 AGD cases. Ribbon-like filaments positive for tau-C antibody were labelled with secondary antibody conjugated to 5 nm gold particles. Scale bar: 50 nm. Ac, nucleus accumbens; Mb, midbrain; Amy, amygdala. Numbers at the beginning indicate the case numbers.

In Western blot analysis, hyperphosphorylated full-length 4R tau (64 and 68 kDa) and strong C-terminal tau bands of 22, 37 (doublets) and 43 kDa were detected in sarkosyl-insoluble fractions of the nucleus accumbens, which were indistinguishable from those of a typical case of AGD in Cases 1, 2, 3 and 5.^12,37,39^ Furthermore, the midbrain fraction showed the same pattern as that in Case 2. Using immunoelectron microscopy, the morphology of tau filaments from the nucleus accumbens in Cases 1, 2, 3 and 5, the amygdala in Case 4 and the midbrain in Case 2 was investigated. Tau-C-positive ribbon-like tau filaments, indicative of AGD,^12,37,39^ were also observed (Fig. 5 and Supplementary Fig. 3 (uncropped blot/gel images)). None of the cases had known variants of the *MAPT* gene.

### Comparative study of DG cases with and without parkinsonism

DG cases with parkinsonism exhibited significantly more argyrophilic grains in the substantia nigra than those without parkinsonism (2.6 ± 0.5 versus 1.4 ± 1.8/2.8 ± 0.4 versus 1.5 ± 0.9; *P* = 0.012/0.025) (lt./rt.), and tended to show more pretangles and GFAs, along with more severe neuronal loss and gliosis. In the limbic system, patients with DG and parkinsonism tended to show a right-dominant distribution of argyrophilic grains. No significant differences were observed in clinical presentation (age at death, duration of cognitive decline, CDR score, MMSE score and HDS-R score) or other comorbid pathologies (CERAD Stage, Thal phase, Braak NFT Stage, BBAR Lewy Stage and BBAR pTDP-43 Stage) and vascular lesions. None of the cases exhibited hippocampal sclerosis (Table 2).

**Table 2:** Comparative study of DG cases with and without parkinsonism

Clinical and imaging findings	DG with parkinsonism (*n* = 6)	DG without parkinsonism (*n* = 14)	*P*-value
Clinical presentation			
Age at death (mean ± SD)	87.0 ± 4.3	85.9 ± 6.0	0.690
Sex (male/female)	3/3	9/5	0.642
Duration of disease (years)	5.2 ± 3.9	4.9 ± 1.9	0.856
CDR	1.8 ± 0.4	1.9 ± 0.6	0.936
MMSE score	17.5 ± 4.5	19.0 ± 4.7	0.644
HDS-R score	16.2 ± 5.1	14.4 ± 2.7	0.622
Imaging findings			
Z-score of the left MTL	1.66 ± 1.03	2.57 ± 1.05	0.283
Z-score of the right MTL	2.84 ± 1.24	3.10 ± 0.67	0.745

Pathological findings		DG with parkinsonism (*n* = 5)	DG without parkinsonism (*n* = 14)	
AGs in the medial temporal lobe				
Saito stage	Lt.	2.0 ± 0.6	2.6 ± 0.5	0.040*
	Rt.	2.6 ± 0.8	2.3 ± 0.8	0.484
Pathologies in the substantia nigra				
SN neuronal loss	Lt	1.2 ± 0.4	0.6 ± 0.6	0.09
	Rt	1.4 ± 0.9	0.6 ± 0.7	0.055
SN AGD stage	Lt.	2.6 ± 0.5	1.4 ± 0.8	0.012*
	Rt.	2.8 ± 0.4	1.5 ± 0.9	0.025*
SN PT stage	Lt.	1.4 ± 0.5	0.9 ± 0.6	0.121
	Rt.	1.4 ± 0.5	0.8 ± 0.7	0.099
SN GFA stage	Lt.	1.0 ± 0.0	0.6 ± 0.5	0.085
	Rt.	1.4 ± 0.5	0.6 ± 0.7	0.055
Comorbid pathology				
Brain weight (g)		1188 ± 165	1215 ± 78	0.654
CERAD stage		0.8 ± 0.7	0.5 ± 0.7	0.470
Thal phase		2.2 ± 1.7	1.5 ± 1.1	0.328
Braak NFT stage		1.8 ± 0.4	1.9 ± 0.3	0.733
BBAR Lewy stage		0.0 ± 0.0	0.1 ± 0.3	0.565
BBAR pTDP43 stage		1.8 ± 0.7	1.0 ± 1.0	0.141
Hippocampal sclerosis (%)		0 (0%)	0 (0%)	1.000
Amyloid angiopathy (%)		4 (80%)	9 (64%)	1.000
Arteriolosclerosis (%)		1 (20%)	2 (14%)	1.000
Lacunar infarcts (%)		2 (40%)	6 (43%)	1.000
Microbleeds (%)		1 (20%)	1 (7.1%)	0.468

AGs, argyrophilic grains; AGD, argyrophilic grain disease; BBAR, Brain Bank for Aging Research; CERAD, Consortium to Establish a Registry for Alzheimer’s Disease; GFA, granular fuzzy astrocyte; HDS-R, the Hasegawa Dementia Scale-Revised; MTL, medial temporal lobe; MMSE, mini-mental scale examination; NFT, neurofibrillary tangle; PT, pretangle; SN, substantia nigra.

^*^ *P* < 0.05. Bolded values indicate statistically significant differences.

## Discussion

This study showed the following: (1) DG cases or cases in which AGD is the sole pathology responsible for dementia, accounted for 4.5% of our Japanese consecutive autopsy cohort, and AGD was associated with cognitive decline, particularly in Saito Stage III cases. (2) Thirty percent of DG cases showed parkinsonism, clinically diagnosed as PSP, PDD or DLB, indicating a new common clinical presentation of AGD. (3) In the DG cases with parkinsonism, anti-dopamine transporter immunostaining showed decreased staining, and more argyrophilic grains were observed in the substantia nigra compared to those for DG cases without parkinsonism, which may be associated with parkinsonism.

### Prevalence and clinical features of AGD and DG cases

In the present study, about half of the patients had AGD (Fig. 1A). This value is slightly higher than that reported by Saito *et al*.^14^ and similar to that reported by Adachi *et al*.^21^ (Table 3). The tendency for the prevalence of AGD to increase with age is consistent across Japanese and European studies, including Ferrer *et al*.^61^ (Supplementary Table 6).

**Table 3:** Prevalence of AGD and DG cases in consecutive autopsy cohort studies

	Average age (years)	Demented cases	Evaluation of AGs	Detection of AGs	Prevalence of AGD cases (%)	Average age of AGD cases (years)	Prevalence of DG cases (%)
Martinez (1997)^24^	61.3 ± 15.8	N/A	Braak	Gallyas	17/300 (5.7%)	77.0 ± 5.1	2/300 (0.7%)
Tolnay (1997)^60^	N/A	N/A	Braak	Gallyas	28/301 (9.3%)	82.5 ± 7.7	4/301 (1.3%)
Braak and Braak (1998)^17^	N/A	N/A	Braak	Gallyas	125/2661 (4.7%)	77.0 ± 5.8	18/2661 (0.7%)
Saito (2004)^14^	80.6 ± 8.9	479/1241 (38.6%)	Saito	Gallyas + AT8	449/1241 (36.2%)	N/A	50/1241 (4.0%)
Togo (2005)^22^	79.9 ± N/A	N/A	Braak	Gallyas	33/359 (8.5%)	83.7 ± 7.4	10/359 (2.8%)
Josephs (2008)^25^	N/A	N/A	Braak	Gallyas + AT8	57/359 (15.8%)	N/A	0/359 (0.0%)
Sabbagh (2009)^27^	N/A	N/A	Braak	Gallyas	80/367 (21.7%)	N/A	N/A
Grinberg (2009)^28^	N/A	N/A	Braak	Gallyas + AT8	36/307 (11.5%)	N/A	1/307 (0.3%)
Nelson (2010)^29^	N/A	N/A	Braak	Gallyas	75/334 (22.5%)	N/A	N/A
Adachi (2010)^20^	81.1 ± 8.9	291/653 (44.6%)	Saito	Gallyas + AT8	304/653 (46.5%)^a^	83.5 ± 8.3^a^	7/653 (1.1%)
Rodriguez (2016)^30^	74.0 ± 11.7	407/983 (41.4%)	Braak	PHF-1	152/983 (15.5%)	78.9 ± 9.4	4/983 (0.4%)
Kovacs (2017)^26^	77.9 ± N/A	319/628 (50.8%)^b^	Braak	AT8	57/628 (9.1%)	79.7 ± N/A	3/628 (0.5%)^c^
Forrest (2022)^31^	83.2 ± N/A	39/101 (38.6%)	Braak	AT8	15/101 (14.9%)	N/A	N/A
Yoshida (2023)^32d^	70.0 ± 14.1	158/1449 (10.9%)	Saito	Gallyas	342/1449 (23.6%)	79.7 ± 9.0	4/1449 (0.3%)
This report	79.3 ± 11.9	164/453 (36.2%)	Saito	Gallyas + AT8	227/444 (51.1%)	83.9 ± 8.5	20/444 (4.5%)

N/A, not available; DG, dementia with grains; AG, argyrophilic grain; AGD, argyrophilic grain disease; Braak, based on Braak’s original description; Saito, based on Saito stage.

^a^Data extracted from our database.

^b^Total number of mild cognitive impairment and demented cases.

^c^Non-demented cases possibly included.

^d^Forensic autopsy cohort.

The frequency of AGD in consecutive autopsy cohorts varied widely from 4.9% to 51.1%, although the mean age, prevalence of dementia and average age of AGD cases were not significantly different among cohorts (Table 3). A possible explanation for this difference is the method used to evaluate AGD. In studies of European,^17,27,32,62^ American^25,26,28,30^ and Brazilian cohorts,^29,31^ the evaluation criteria for AGD were based on the accumulation of argyrophilic grains in the transentorhinal area and hippocampal CA1, as originally described by Braak *et al*.,^1^ which corresponds to Saito Stage III and part of Saito Stage II.^14^ Another reason is the variation in the sensitivity of immunostaining or Gallyas staining among facilities. The sensitivity of Gallyas staining can vary widely among institutions, and RD4 staining shows different sensitivities depending on the tissue fixation conditions.^21^ In AT8 staining, argyrophilic grains are often difficult to differentiate from neuropil threads when concomitant Alzheimer’s pathology is severe.^63^

Our study showed a high prevalence of DG cases, accounting for 4.5% (20/444) of Japanese consecutive autopsy cases. This finding is consistent with previous reports on Japanese autopsy cohorts. In addition to the study conducted by Saito *et al*.,^14^ Togo *et al*. reported 10 DG cases (2.6%) among 386 consecutive autopsy cases in another Japanese cohort of patients from a psychiatric hospital.^23^ Furthermore, in 2010, Adachi *et al*. reported another seven cases (1.1%) among 653 consecutive autopsy cases from our cohort.^21^ Among European cohort-based studies, Tolnay reported four cases (1.3%) among 301 consecutive autopsy cases.^62^ However, other studies from European, American and Brazilian cohorts found DG cases in <1% of large autopsy cohorts or reported no DG cases, despite rigorous research,^25-32^ including Braak and Braak’s large cohort study, which reported 16 cases (0.7%) among 2661 cases.^17^ (Table 3) There were several reasons for this finding. Firstly, the definition of the DG, namely that AGD is the ‘sole pathology responsible for dementia’, depends on the criteria regarding how other comorbid pathologies could affect cognitive decline, which is not clarified in previous reports. Further studies with uniform criteria are needed to evaluate the presence or absence of AGD and the extent to which other comorbid pathologies contribute to cognitive dysfunction.

A significant association has been observed between AGD and cognitive decline. In addition to several cohort studies,^14,21,23^ the association between AGD and cognitive decline is further supported by the frequent observation of AGD in mild cognitive impairment cases,^64^ PDD cases^65^ and centenarians,^63^ and AGD was recently shown to be a risk factor for cognitive decline in cases with less than Braak Stage IV.^36^ Interestingly, when examined for each Saito Stage, Stage I patients showed a significant association, but Stage II patients did not show an association with cognitive decline and prevalence of dementia (Table 1). This may be due to the presence of comorbid neurodegenerative diseases. For Saito Stage III cases, we demonstrated an association with cognitive decline and prevalence of dementia, even in age-, sex- and comorbid AD pathology-matched cases (Table 1). The same trend was observed in a study of pure AGD cases. Approximately 70% of Saito Stage III pure AGD cases were demented, and 80% had some cognitive decline, consistent with our previous report.^14^ Conversely, only 10% of Saito Stage II pure AGD cases were demented, and 20% of cases had some cognitive decline. No cases of Saito Stage I pure AGD patients were demented, and only 7% had some cognitive decline (Fig. 1B). This finding indicates that Saito Stage should be considered when assessing the association between cognitive decline and AGD.

Our study also showed that an antemortem diagnosis of DG is possible if thorough clinical and imaging studies are performed. Four out of five such cases are diagnosed based on imaging findings, namely anterior-dominant limbic-predominant atrophy compared to the neocortical areas,^66,67^ often accompanied by asymmetry,^21^ in addition to slowly progressive memory disturbance. For syndromes indicating frontotemporal dementia, including irritability, dysregulation and apathy,^68^ the prevalence is not very high, accounting for only one-third of DG cases. Imaging studies are more useful for diagnosing DG, and further autopsy-based studies are necessary for a more reliable antemortem diagnosis.

### Parkinsonism is a new common clinical feature of AGD

The association between AGD and parkinsonism has been reported in several case reports^34,35^ and cohort-based studies.^26,29^ Furthermore, patients carrying *MAPT* S305S variants show AGD-like pathology and parkinsonism.^69^ In addition, several AGD cases with extensive pathology involving the basal ganglia and neocortex have been reported to present with parkinsonism^36,70-72^ or gait impairment.^73^ However, these cases have been reported to exhibit extensive or exceptional clinical presentations of AGD.

The prevalence of parkinsonism in DG cases was 30% in our consecutive autopsy cohort. This is comparable to the prevalence of frontotemporal signs. More interestingly, one case presented in this study showed parkinsonism even at Saito stage II, in which argyrophilic grains in the limbic system were moderately extended. The relatively high prevalence of parkinsonism is not restricted to our study. Josephs *et al*. identified one case of parkinsonism and two cases with gait impairment among eight cases of AGD with cognitive decline, although clinical details were not described.^26^ Parkinsonism and gait impairment are common clinical features of AGD.

The six DG cases presenting with parkinsonism accounted for 10% of cases with parkinsonism and cognitive decline, rising to 13.9% among patients over 80 years old in our consecutive autopsy cohort. Similarly, Glimberg and Heinsen reported that AGD cases accounted for 14.3% of cases presenting with parkinsonism.^29^ AGD is an emerging differential diagnosis for elderly patients presenting with cognitive decline and parkinsonism, including DLB, PSP and normal pressure hydrocephalus.

Parkinsonism, observed in six DG cases, was characterized by postural instability followed by mild rigidity. Akinesia and tremors were not prominent features in these cases. In contrast to Lewy body disease, autonomic dysfunction, REM sleep disorder and olfactory dysfunction were not observed. Additionally, L-Dopa therapy resulted in minimal clinical improvement. Furthermore, although all six cases were demented at autopsy, parkinsonism preceded cognitive decline in Cases 2 and 4. This finding indicates that in elderly individuals, AGD may present with symptoms resembling Parkinson’s disease and PSP without cognitive decline.

On brain MRI, anterior predominant atrophy of the medial temporal lobe was conspicuous among Saito Stage III cases (Cases 1–4), and asymmetrical atrophy was observed in two cases (Cases 2 and 3), consistent with the findings of typical AGD cases.^21,66,67^

### Pathological background of DG cases presenting with parkinsonism

Several studies have examined anti-phosphorylated tau immunoreactive structures in the substantia nigra and parkinsonism in patients with Alzheimer’s disease without Lewy body pathology. The presence of such cases was first reported by Morris *et al*.^74^ Liu *et al*. reported that the amount of AT8 immunoreactive structures and neuropil threads in the substantia nigra is associated with parkinsonism.^75^ Similarly, Burns *et al*. showed that parkinsonism is associated with anti-phosphorylated tau immunoreactive structures in the substantia nigra.^76^ Furthermore, Murray *et al*. reported reduced dopamine transporter immunoreactivity in the basal ganglia,^77^ indicating decreased nigrostriatal function in studies involving Parkinson’s disease cases,^78-80^ despite the preserved density of melanin-containing neurons in the substantia nigra. Schneiders *et al*.^81^ and Chu *et al*.^82^ showed that anti-phosphorylated tau immunoreactive structures are observed not only in patients with Alzheimer’s disease but also in normal elderly individuals and are associated with parkinsonism, especially gait impairment. A recent study has suggested tau astrogliopathy in the substantia nigra and its possible effects on neuronal dysfunction.^83^ Another recent study demonstrated an association between Saito Stage and neuronal loss in the substantia nigra, as well as between severe argyrophilic grains and the basal ganglia.^36^ Considering these results, they concluded that the nigrostriatal system was inhibited by the accumulation of phosphorylated tau immunoreactive structures in the substantia nigra. The mechanism has not been clarified, but it is considered to differ from the neuronal loss in the substantia nigra that underlies parkinsonism.^76,77^

Among the six DG cases presenting with parkinsonism, DAT-SPECT was performed in two cases, both of which had decreased uptake in the putamen. Neuropathological examination revealed that phosphorylated tau immunoreactive structures in the substantia nigra involve argyrophilic grains, pretangles and GFAs, and were immunoreactive for anti-four-repeat tau immunostaining, but not for three-repeat tau immunostaining. Therefore, they are indicative of AGD pathology, as confirmed by Western blotting and immunoelectron study in Case 2. The number of argyrophilic grains was significantly greater in patients with parkinsonism than in those without parkinsonism. Other structures, including pretangles and GFAs, were also larger, although not significantly. Furthermore, decreased anti-dopamine transporter immunostaining is consistent with decreased dopamine levels in the caudate and putamen in two AGD cases with extensive pathology reported by Yamada *et al*.,^73^ indicating reduced nigrostriatal system activity in the basal ganglia of AGD cases. Therefore, we consider that AGD pathology in the substantia nigra is associated with parkinsonism, and the mechanism involves inhibition of the nigrostriatal system, which may be different from other four-repeat tauopathies, including CBD or PSP, due to neuronal loss in the substantia nigra, but similar to what is observed in Alzheimer’s disease cases.

### Limitations

This study had a few limitations. Firstly, cases of DG presenting with parkinsonism may be rare, accounting for 1% of our autopsy cohort. Considering the relatively low percentage of DG cases in the European, American and Brazilian cohorts, similar cases may not be found and may instead be considered a ‘rare presentation of AGD’ rather than a distinct disease entity. The clinical presentation and rigorous pathological research of similar cases are necessary. Secondly, the four-repeat tau immunoreactive structures in the substantia nigra may not be an extension of AGD but rather part of other overlapping neurodegenerative diseases. We could not completely rule out this possibility, but it is more reasonable to speculate that AGD pathology underlies both the limbic and nigrostriatal systems, as shown in Case 2, rather than considering it incidental, given that the same concomitant pathology overlaps in the five cases. Another limitation is that we evaluate the more severe side for LATE-NC, which is often asymmetric. Although when neuronal cytoplasmic inclusions were observed, we evaluated the contralateral side, this approach may lead to an underestimation of the other side with early-stage LATE-NC.

## Conclusion

In conclusion, AGD has a strong association with cognitive decline, especially in Saito Stage III cases. DG cases account for a certain proportion of consecutive autopsy cohorts and are not rare clinical presentations of AGD. Parkinsonism is a common clinical presentation of DG. The extension of AGD to the nigrostriatal system may contribute to the development of parkinsonism.

## Supplementary Material

fcaf352_Supplementary_Data

## Data Availability

No new software and/or algorithms, in-house scripts, programmes or codes were generated to support this study. The data that support the findings of this study are available on request from the corresponding author. The data are not publicly available due to privacy or ethical restrictions.
