# Supplementary material for: Argyrophilic grain disease: epidemiology and association with cognitive decline and parkinsonism
Source: Brain Commun. 2025 Oct 13;7(5):fcaf352. doi: 10.1093/braincomms/fcaf352 (PMC12516700; doi:10.1093/braincomms/fcaf352)
Supplement: fcaf352_Supplementary_Data [file fcaf352_supplementary_data.pdf]

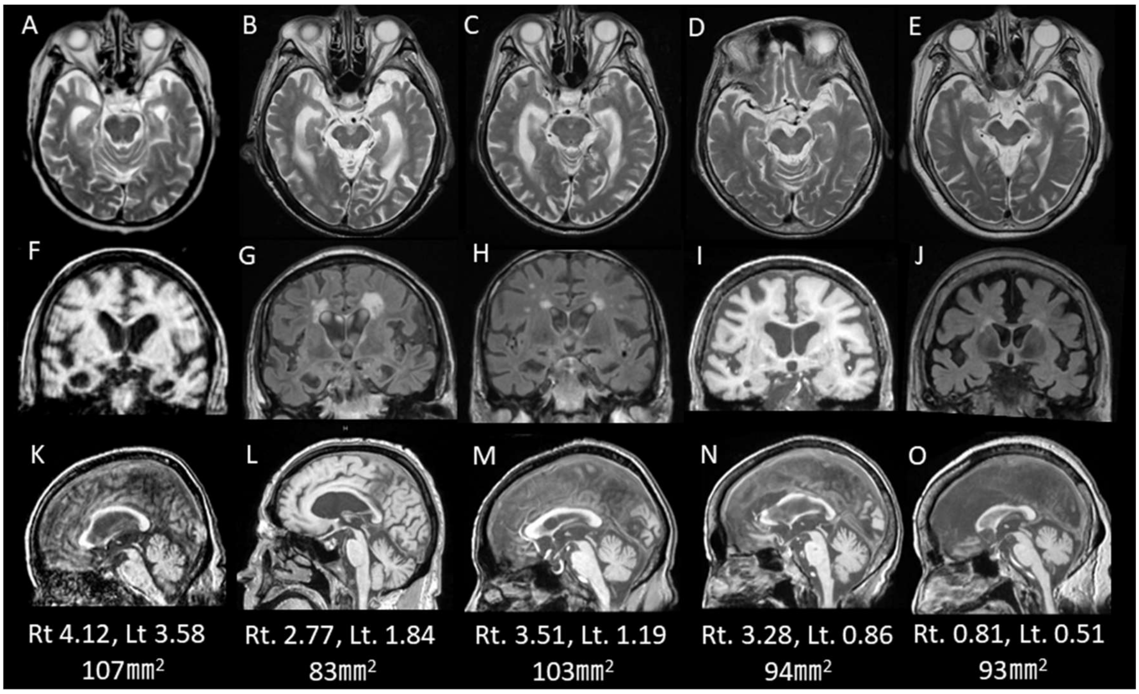

Supplementary Figure.1 Magnetic resonance imaging findings of dementia with grains cases with parkinsonism

Atrophy of medial temporal lobe in coronal (**A**–**E**) and sagittal (**F**–**J**) section. Atrophy of midbrain tegmentum was not prominent (**K**–**O**). (**A**–**E**)：T2 weighed image, (**F**)(**I**), (**K**-**O**)：T1 weighed image, (**G**)(**H**)(**J**)：FLAIR image. The upper number at the bottom of the figure indicates the Z-score indicating medial temporal lobe atrophy, and the lower number indicates the area of the midbrain tegmentum.

(**A**)(**F**)(**K**): case 1, (**B**)(**G**)(**L**): case 2, (**C**)(**H**)(**M**): case 3, (**D**)(**I**)(**N**): case 4, (**E**)(**J**)(**O**): case 5.

FLAIR: fluid attenuated inversion recovery

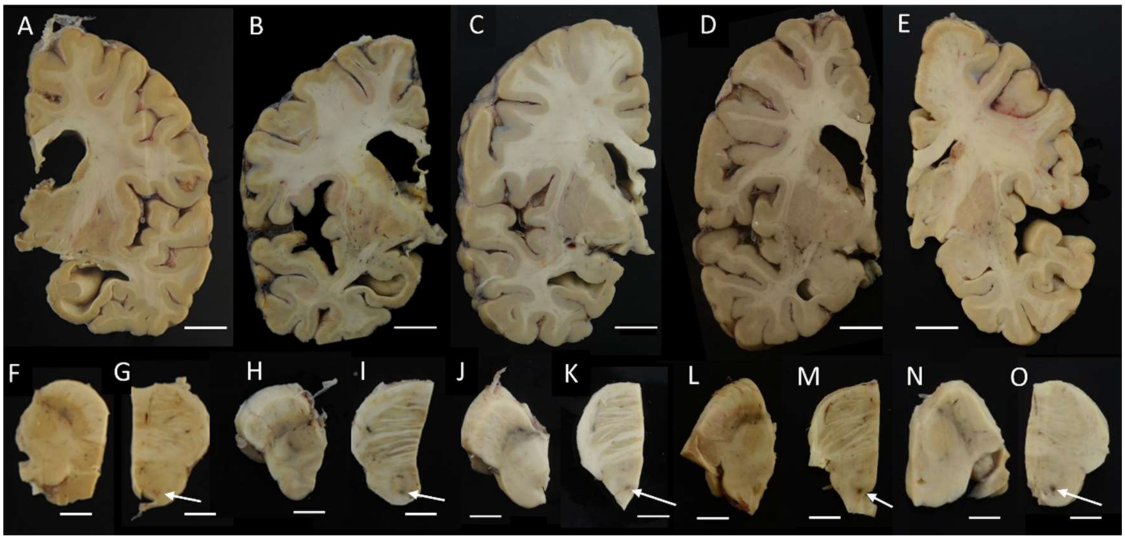

Supplementary Figure.2 Macroscopic findings of dementia with grains with parkinsonism

Severe (**A-C**), moderate (**D**), and mild (**E**) atrophy of the amygdala.

Mild (**F**) and minimal(**H, J, L, N**) depigmentation of substantia nigra.

Depigmentation of the locus coeruleus was not conspicuous (arrows). ((**G**)(**I**)(**K**)(**M**)(**O**))

(**A, F, G**): case 1, (**B, H, I**): case 2, (**C, J, K**): case 3, (**D, L, M**): case 4, (**E, N, O**): case 5. Scalebars：(**A**)-(**E**): 2 cm, (**F**)-(**O**): 1 cm

Supplementary Figure 3. Source images of immunoblots for Fig. 5A

Case 1 (T46)

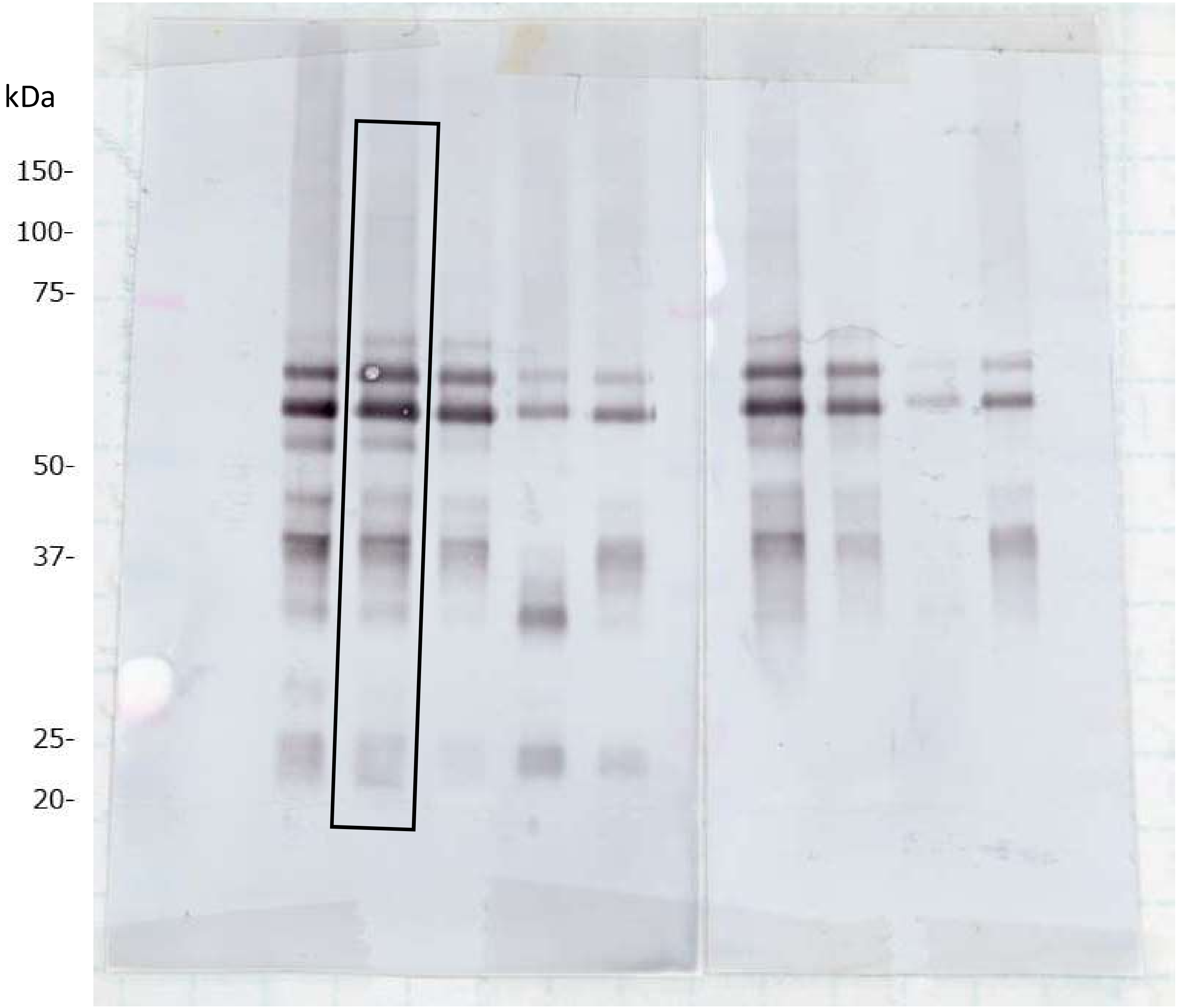

Case 2 (T46)

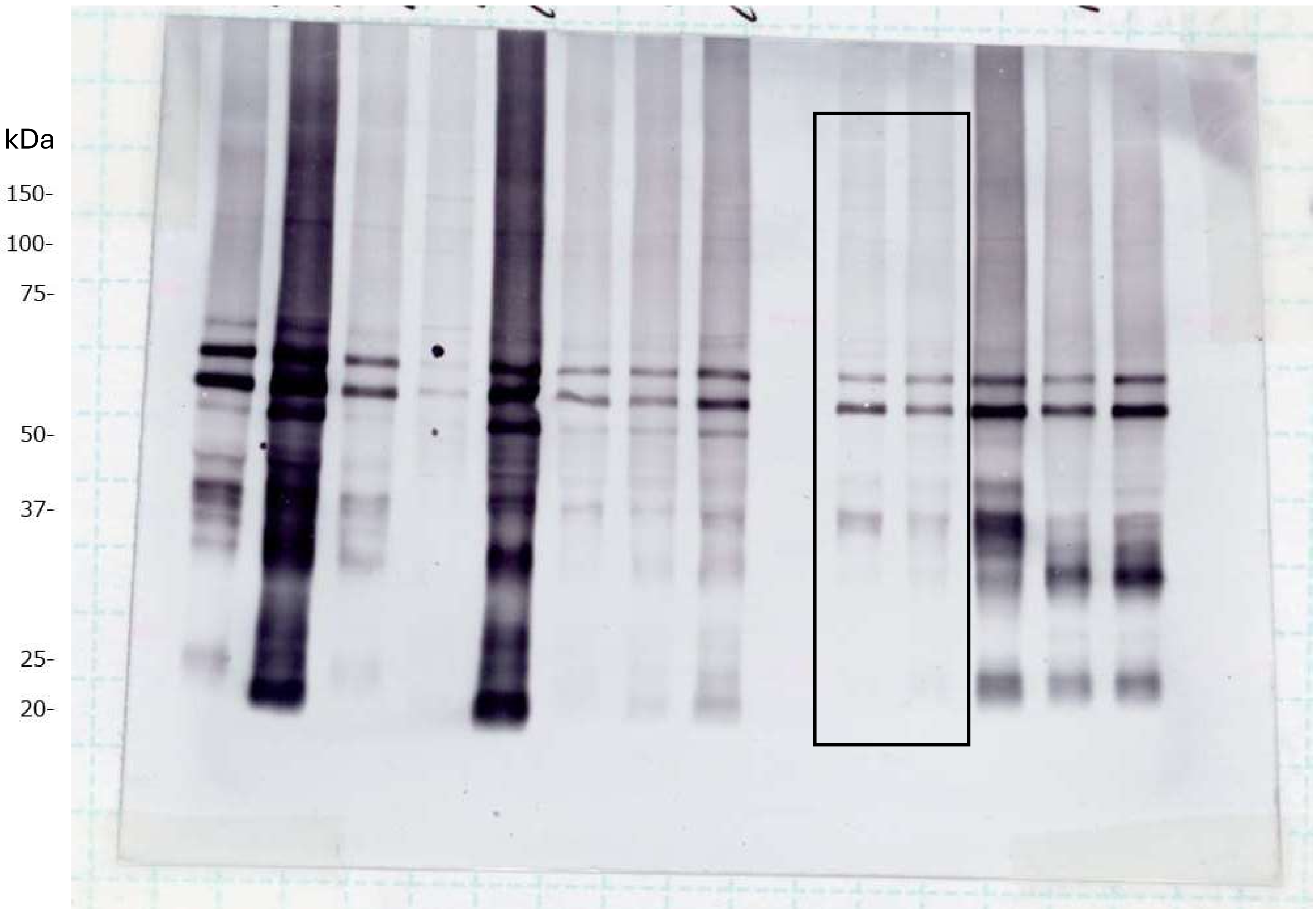

Case 3 (T46)

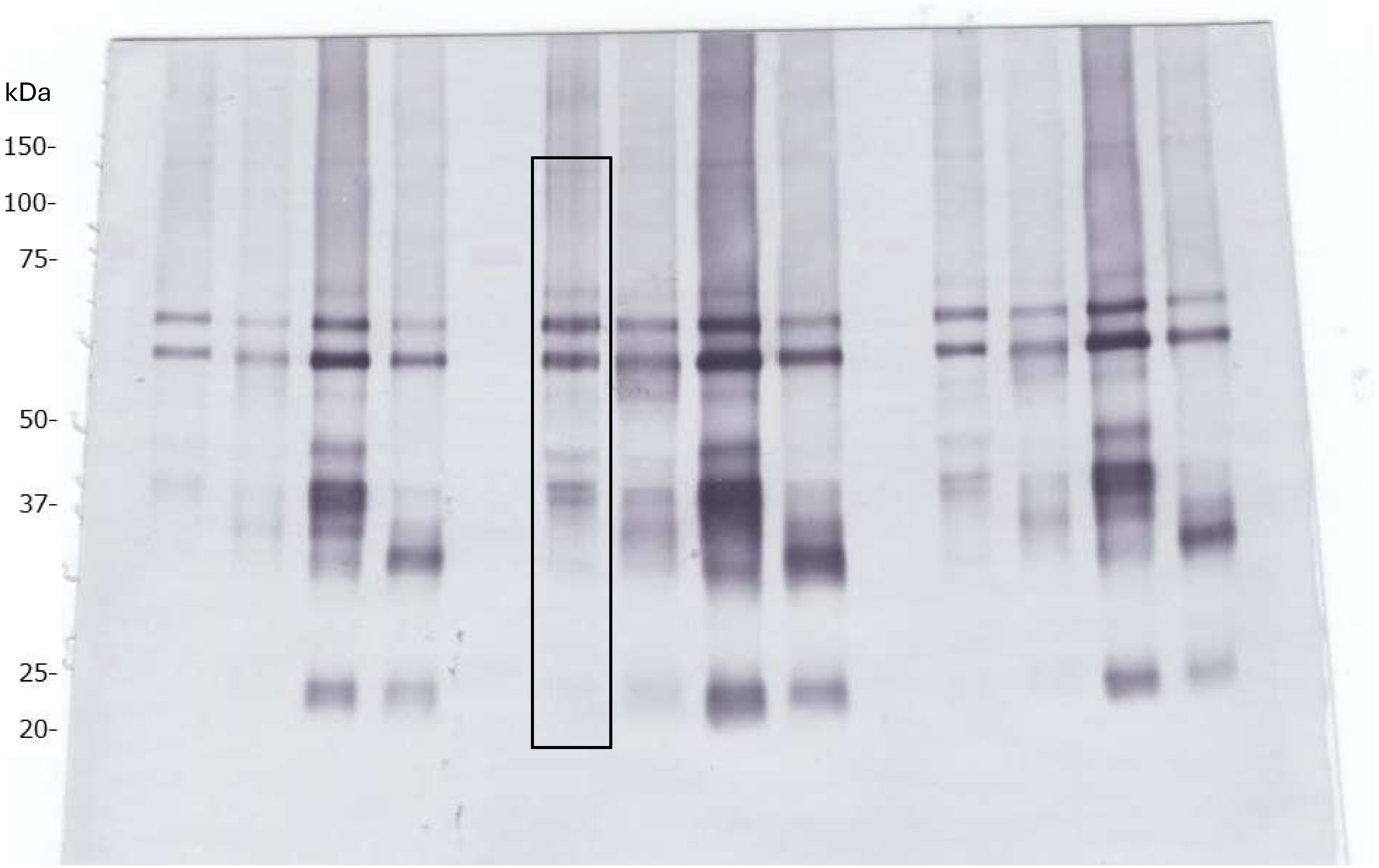

Case 5 (tau-C)

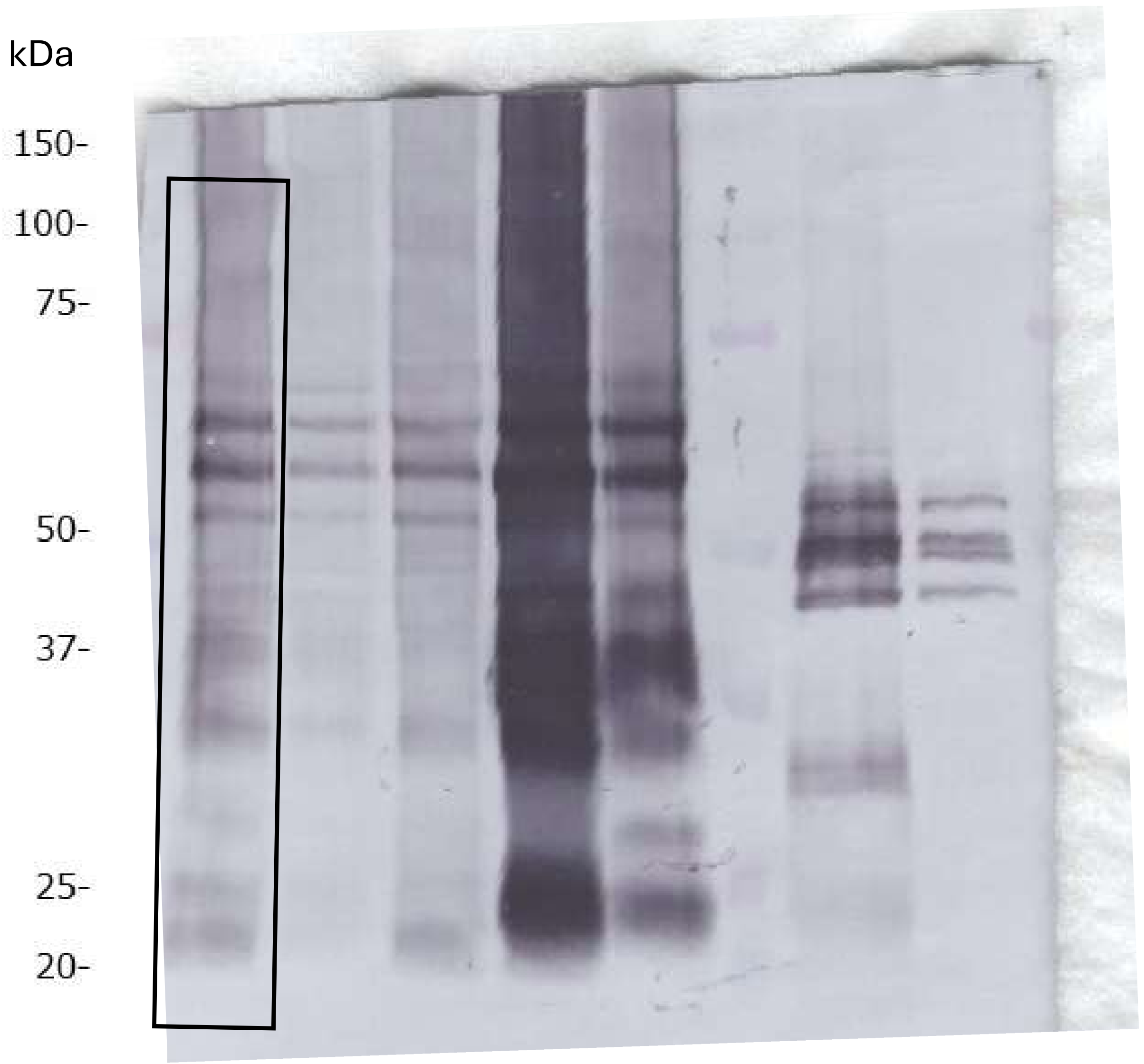

Supplementary Table 1. Regression analyses of predictors for clinical dementia rating score and the presence of dementia

Supplementary Table 1-1. Multivariate analyses of predictors for clinical dementia rating score

	β	t	p value	VIF
Residuals		1.158	0,247	
Age	-0.009	-1.289	0.198	1.36
Sex	-0.141	-1.197	0.234	1.07
AGD Saito stage	0.129	2.274	**0.024***	1.16
BBAR pTDP43	0.224	3.912	**< 0.001*****	1.21
Thal	0.03	0.723	0.469	1.51
Braak NFT	0.317	5.476	**< 0.001*****	1.72
BBAR Lewy	0.125	2.719	**0.006****	1.1

Supplementary Table 1-2. Logistic regression analysis for the presence of dementia

	Odds ratio	95% CI	p value	VIF
Age	0.998	0.97-1.03	0.921	1.288
Sex	0.721	0.43-1.21	0.148	1.041
AGD Saito stage	1.49	1.16-1.90	**0.00152****	1.127
BBAR pTDP43	1.53	1.19-1.97	**< 0.001*****	1.071
Thal	1.07	0.89-1.27	0.476	1.327
Braak NFT	1.83	1.37-2.44	**< 0.001*****	1.447
BBAR Lewy	1.44	1.14-1.82	**< 0.001*****	1.037

$^{*}p < 0.05$, $^{**}p < 0.01$, $^{***}p < 0.001$ Bolded values indicate statistically significant differences.

AD: Alzheimer disease, AGD: argyrophilic grain disease, BBAR: brain bank for aging research, NFT: neurofibrillary tangle

Supplementary Table 2. Extraction of pure argyrophilic grain disease

452 consecutive autopsy cases

↓

Excluded (n = 225)
- Cases without AGD (n = 217)
- Severe brain damage (n = 8)

AGD positive cases (n = 227)

↓

Other neurodegenerative disease (n = 83)
- Alzheimer disease (n = 21)
- Neurofibrillary tangle predominant dementia(n = 10)
- Lewy body disease* (n = 18)
- Amyotrophic lateral sclerosis (n = 11)
- Frontotemporal lobar degeneration (n = 3)
- Progressive supranuclear palsy (n = 9)
- Corticobasal degeneration (n = 4)
- Creutzfeldt-Jakob disease (n = 4)
- Other neurodegenerative disease (n = 3)

Cases with othe senile changes and conditions that induce cognitive decline (n = 45)
- Vascular dementia (n = 9)
- Brain tumor (n = 5)
- Extensive LATE-NC*** (n = 5)
- Braak NFT stage ≧ III (n=21)
- prodromal Lewy body disease** (n = 5)

↓

Pure argyrophilic grain disease (n = 99)

* Cases with BBAR Lewy stage ≧3

** Cases with BBAR Lewy stage =2

*** more than 20 neuronal cytoplasmic inclusions per lower field

AGD: argyophilic grain disease, BBAR: brain bank for aging research, LATE-NC: limbic age dependent TDP-43 encephalopathy neuropathological change, NFT: neurofibrillary tangle

Supplementary Table 3. Clinical presentations of DG cases presenting with parkinsonism and without parkinsonism

Case	Age at death (years)	Sex	Age at onset (years)	Disease duration (years)	Cognitive decline duration (years)	Pism duration (years)	Clinical diagnosis	CDR	MMSE	HDS-R	Doinant side	Onset Symptoms	Postural instability	Truncal rigidity	Rigidity of extremities	Tremor	Akinesia
**DG with parkinsonism**																	
1	85	M	82	3	3	2	PSP+AD	2	12	7	equivalent	memory disturbance	3	1	0	0	1
2	85	M	74	11	4	11	PSP	2	21	16	equivalent	gait instability	3	0	0	0	0
3	84	M	82	2	2	2	DLB	1	24	21	equivalent	memory disturbance, postural instability	2	0	1	0	0
4	93	F	84	9	3	9	PDD	2	18	21	right	gait instability	2	0	1	0	0
5	92	F	90	2	2	2	DLB	2	16	14	right	memory disturbance, postural instability	2	0	1	1	0
6	83	F	79	4	4	3	PSP+AD	2	14	N/A	equivalent	memory disturbance	2	0	1	1	1
**DG without parkinsonism**																	
7	79	M	73	6	6		Dem	2	N/A	18							
8	75	M	N/A	N/A	N/A		Dem	2	N/A	N/A							
9	81	M	79	2	2		Dem	1	21	N/A							
10	90	M	N/A	N/A	N/A		Dem	2	N/A	N/A							
11	81	F	77	4	4		DG	2	25	13							
12	96	F	N/A	N/A	N/A		Dem	1	N/A	N/A							
13	95	M	N/A	N/A	N/A		DG	2	N/A	N/A							
14	90	M	84	6	6		DG	3	11	11							
15	83	M	80	3	3		Dem	1	N/A	N/A							
16	86	M	80	6	6		DG	2	21	17							
17	81	F	73	8	8		AD	1	N/A	N/A							
18	85	M	79	6	6		Dem	3	N/A	N/A							
19	92	F	N/A	N/A	N/A		Dem	2	N/A	N/A							
20	88	F	85	3	3		DG	2	17	13							

AD: Alzheimer's disease, CDR: clinical dementia rating, Dem: dementia but without thorough examination, DLB: dementia with Lewy bodies, HDS-R: the Hasegawa dementia scale-revised, MMSE: mini-mental scale examination, N/A: not assessed, Pism: parkinsonism, PDD: Parkinson disease with dementia, PSP: progressive supranuclear palsy

Supplementary Table 4. AGD pathology in the medial temporal lobe and nigrostriatal system and comorbid pathologies of DG cases presenting with parkinsonism and without parkinsonism

			**AGs in MTL**		**Pathologies in substantia nigra**								**Comorbid pathologies**											
Case		Sex	Saito Stage		SN neuronal loss		SN AGD stage		SN PT stage		SN GFA stage		Brain weight (g)	CERAD stage	Thal phase	Braak NFT stage	BBAR Lewy stage	BBAR pTDP-43 stage	Hippocampal sclerosis	Amyloid angiopathy	Arteriolo sclerosis	Lacunar infarcts	Micro bleeds
**DG with parkinsonism**			Lt.	Rt.	Lt.	Rt.	Lt.	Rt.	Lt.	Rt.	Lt.	Rt.											
1	85	M	3	3	1	2	3	3	2	2	1	2	1297	B	5	2	0	3	0	1	0	0	0
2	85	M	2	3	1	2	3	3	1	2	1	2	1307	A	2	2	0	2	0	1	0	0	0
3	84	M	2	3	1	1	2	3	2	1	1	1	1299	none	0	1	0	1	0	0	0	0	0
4	93	F	1	3	2	2	3	3	1	1	1	1	1159	none	1	2	0	1	0	1	1	1	1
5	92	F	2	1	1	0	2	2	1	1	1	1	878	none	1	2	0	1	0	1	0	1	0
**DG without parkinsonism**																							
7	79	M	3	3	0	1	2	2	1	1	1	2	1148	none	1	1	0	0	0	0	1	1	1
8	75	M	3	1	0	0	0	0	0	0	0	0	1280	none	1	2	0	0	0	1	0	0	0
9	81	M	3	3	0	0	2	3	2	2	1	2	1193	none	1	2	0	1	0	0	0	1	0
10	90	M	3	1	0	0	1	1	0	0	0	0	1291	none	1	2	0	1	0	1	1	1	0
11	81	F	2	3	1	0	0	1	0	0	0	0	1300	none	0	2	0	0	0	1	0	0	0
12	96	F	2	3	2	1	3	3	2	2	1	1	1071	B	2	2	0	3	0	1	0	1	0
13	95	M	2	3	1	2	1	2	1	1	0	0	1274	A	3	1	0	1	0	1	0	0	0
14	90	M	3	3	1	1	1	1	1	1	1	1	1189	A	3	2	0	1	0	1	0	0	0
15	83	M	3	3	1	1	2	3	1	1	1	1	1300	none	1	2	0	1	0	1	0	0	0
16	86	M	3	2	0	N/A	1	N/A	1	N/A	1	N/A	1194	none	1	2	0	0	0	0	0	1	0
17	81	F	3	2	1	1	2	1	1	1	1	1	1053	A	3	2	0	1	0	1	0	0	0
18	85	M	3	1	1	1	2	1	1	0	0	0	1283	none	0	2	0	0	0	0	0	1	0
19	92	F	2	2	0	0	1	1	0	0	0	0	1227	none	1	2	0	2	0	0	0	0	0
20	88	F	2	2	1	0	2	1	1	1	1	0	1209	A	3	2	1	3	0	1	0	0	0

AD: Alzheimer's disease, AG: argyrophilic grain, AGD: argyrophilic grain disease, BBAR: brain bank for aging research, CERAD: Consortium to Establish a Registry for Alzheimer's Disease,
GFA: granular fuzzy astrocyte, MTL: medial temporal lobe, NFT: neurofirbillary tangle, N/A: not assessed, PT: pretangle, SN: substantia nigra,

Supplementary Table 5. Comparative study of dementia with grains cases with parkinsonism and Dat normal control

	DG with parkinsonism (n = 5)	DAT normal control (n = 5)	p-value
Area ratio	0.63 ± 0.09	0.81 ± 0.07	0.0159*
Age at death (mean ± SD)	87.8 ± 3.9	68.6 ± 15.2	0.0593
Sex (Male/Female)	3/2	3/2	1.000
Brain weight (g)	1188 ± 165	1265 ± 124	0.841
AGD Saito stage	2.8 ± 0.4	1.0 ± 0.6	0.0118*
CERAD stage	0.8 ± 0.7	2.0 ± 1.1	0.131
Thal phase	2.2 ± 1.7	3.4 ± 1.7	0.395
Braak NFT stage	1.8 ± 0.4	3.2 ± 1.7	0.329
BBAR Lewy stage	0.0 ± 0.0	0.3 ± 0.4	0.160
BBAR pTDP43 stage	1.8 ± 0.7	1.2 ± 0.7	0.373

* p<0.05

AGD: argyrophilic grain disease, BBAR: brain bank for aging research, CERAD: Consortium to Establish a Registry for Alzheimer's Disease,

DAT: dopamin transporter, DG: dementia with grains, NFT: neurofibrillary tangle,

Supplementary Table 6. Prevalance of AGD cases in different age categories

Prevalence of each age group	Under 60 years	60-69 years	70-79 years	Over 79 years
Saito (2004)[14]	0 % (0/14)	17.6 % (19/108)	31.2 % (132/423)	42.8 % (298/696)
Ferrer (2008)[59]	10 % (N/A)<br>*Under 61 years	17 % (N/A)<br>*61-70 years	30 % (N/A)<br>*71-80 years	43 % (N/A)<br>*Over 80 years
Adachi (2010)[20]**	0 % (0/9)	14.0 % (6/43)	39.1 % (92/235)	56.2 % (205/365)
Yoshida (2023)[32]	2.9 % (10/346)	11.9 % (35/294)	26.1 % (97/371)	45.7 % (200/438)
This report	0 % (0/26)	31.8 % (14/44)	43.8 % (49/112)	62.7 % (165/263)

** data extracted from our database
